# Detecting shrub encroachment in seminatural grasslands using UAS LiDAR

**DOI:** 10.1002/ece3.6240

**Published:** 2020-05-06

**Authors:** Bjarke Madsen, Urs A. Treier, András Zlinszky, Arko Lucieer, Signe Normand

**Affiliations:** ^1^ Section for Ecoinformatics & Biodiversity Center for Biodiversity Dynamics in a Changing World Department of Biology Aarhus University Aarhus C Denmark; ^2^ MTA Centre for Ecological Research Tihany Hungary; ^3^ Discipline of Geography and Spatial Sciences University of Tasmania Hobart Australia

**Keywords:** biomass, grassland dynamics, remote sensing, scotch broom, shrub encroachment, UAS LiDAR

## Abstract

1. Shrub encroachment in seminatural grasslands threatens local biodiversity unless management is applied to reduce shrub density. Dense vegetation of *Cytisus scoparius* homogenizes the landscape negatively affecting local plant diversity. Detecting structural change (e.g., biomass) is essential for assessing negative impacts of encroachment. Hence, exploring new monitoring tools to achieve this task is important for effectively capturing change and evaluating management activities.
2. This study combines traditional field‐based measurements with novel Light Detection and Ranging (LiDAR) observations from an Unmanned Aircraft System (UAS). We investigate the accuracy of mapping *C. scoparius* in three dimensions (3D) and of structural change metrics (i.e., biomass) derived from ultrahigh‐density point cloud data (>1,000 pts/m^2^). Presence–absence of 12 shrub or tree genera was recorded across a 6.7 ha seminatural grassland area in Denmark. Furthermore, 10 individuals of *C. scoparius* were harvested for biomass measurements. With a UAS LiDAR system, we collected ultrahigh‐density spatial data across the area in October 2017 (leaf‐on) and April 2018 (leaf‐off). We utilized a 3D point‐based classification to distinguish shrub genera based on their structural appearance (i.e., density, light penetration, and surface roughness).
3. From the identified *C. scoparius* individuals, we related different volume metrics (mean, max, and range) to measured biomass and quantified spatial variation in biomass change from 2017 to 2018. We obtained overall classification accuracies above 86% from point clouds of both seasons. Maximum volume explained 77.4% of the variation in biomass.
4. The spatial patterns revealed landscape‐scale variation in biomass change between autumn 2017 and spring 2018, with a notable decrease in some areas. Further studies are needed to disentangle the causes of the observed decrease, for example, recent winter grazing and/or frost events.
5. *Synthesis and applications:* We present a workflow for processing ultrahigh‐density spatial data obtained from a UAS LiDAR system to detect change in *C. scoparius*. We demonstrate that UAS LiDAR is a promising tool to map and monitor grassland shrub dynamics at the landscape scale with the accuracy needed for effective nature management. It is a new tool for standardized and nonbiased evaluation of management activities initiated to prevent shrub encroachment.

## Graphical abstract

We developed a workflow for detecting shrub encroachment by *Cytisus scoparius* in a seminatural Danish grassland. We use highly detailed spatial information from UAV LiDAR point clouds to recognize shrub genera and estimate biomass between two years of monitoring.

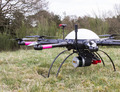

## 1 INTRODUCTION

Plant diversity in natural and seminatural grasslands experiences severe pressure from shrub encroachment (Timmermann, Damgaard, Strandberg, & Svenning, 2015; Wilsey, 2018). Establishment of woody species is a successional stage gradually occurring in grasslands and may induce encroachment if not exposed to natural processes, such as browsing, trampling and fires, or if grasslands are not managed otherwise. (D'Odorico, Okin, & Bestelmeyer, 2012). Shrub encroachment is enhanced by nutrient enrichment (Stevens, Dise, Mountford, & Gowing, 2004) and land‐use abandonment (Deák, Valkó, Török, & Tóthmérész, 2016) that leads to a decrease in plant species richness unless management strategies are implemented to keep shrub dominance low (Kesting, Petersen, & Isselstein, 2015). Grassland plant diversity is generally highest at intermediate levels of biomass (Ejrnæs & Bruun, 2000), and this relationship is also found for shrub encroachment (Kesting et al., 2015). High density of *Cytisus scoparius* is known to homogenize the landscape and to decrease plant diversity, especially in grasslands (Bellingham, 1998). The presence of *C. scoparius* can favor the establishment of fast‐growing species by decreasing the soil C:N ratio and hereby increasing N availability (Haubensak & Parker, 2004). *Cytisus scoparius* vegetation is therefore considered problematic to grasslands including many areas within its European native range (Sheppard, Hodge, Paynter, & Rees, 2002). The number of *C. scoparius* individuals in an area is not necessarily representative for invasiveness, and Parker (2000) suggests that structural changes in *C. scoparius* (e.g., biomass and density) are important to assess the negative impact on plant diversity. Hence, the structural components are necessary for evaluating the risk of grassland plant diversity decline resulting from encroachment and to assess the efficiency of management activities. Monitoring efforts that highlight the need for action (e.g., shrub reduction) should be strategically aligned with the actual spatial scale of management (Magurran, 2016). However, monitoring shrub dynamics with the detail (i.e., small grain size) and spatial extent needed for management is challenging (Cao, Liu, Cui, Chen, & Chen, 2018).

Remote sensing covers larger areas than classical field assessments of structural change, which are time‐consuming and provide only local information (Wachendorf, Fricke, & Möckel, 2017). Satellite‐based spectral information can provide valuable information on cover of *C. scoparius* at the landscape scale for areas with high shrub densities, although limited to observations in the flowering period (Hill, Prasad, & Leckie, 2016). However, the relatively low resolution of satellite imagery (Aplin, 2005) does not allow the quantification of structural information, for example, height and biomass, from individuals or lower concentrated areas of shrubs. In contrast, observations from cameras or LiDAR (Light Detection And Ranging) sensors mounted on an Unmanned Aircraft System (UAS, also referred to as UAV or drone) can provide very high‐resolution information on canopy (Wallace, Lucieer, Malenovský, Turner, & Vopěnka, 2016) or vegetation structure (Forsmoo, Anderson, Macleod, Wilkinson, & Brazier, 2018; Wang et al., 2017).

LiDAR technology is a remote sensing method providing three‐dimensional (3D) point cloud information suitable for quantifying vegetation structure (Lefsky, a, Cohen, W. B., Parker, G. G., & Harding, D. J., 2002). Airborne LiDAR has been used to map grassland vegetation (Zlinszky et al., 2014) and to explain variation in species diversity across varying vegetation communities (Moeslund et al., 2019). Especially, forestry research has successfully implemented airborne LiDAR data to, for example, detect the composition of gymnosperm species in plantations (Donoghue, Watt, Cox, & Wilson, 2007), to evaluate ecosystem services (Vauhkonen, 2018) and management efforts based on forest structure (Valbuena, Eerikäinen, Packalen, & Maltamo, 2016), or to demonstrate how LiDAR can be used to measure vegetation height of sagebrush (Mitchell et al., 2011). Furthermore, stages of shrub encroachment and biomass estimates have been mapped on a coarser resolution (30 m raster) based on a LiDAR point density of 5.6 points/m^2^ (Sankey, Shrestha, Sankey, Hardegree, & Strand, 2013). With advancing technology, it is now possible to mount good quality scanners on UAS (Manfreda et al., 2018). UAS LiDAR systems provide new opportunities to provide ultrahigh point density (>1,000 pts/m^2^) on demand; hence, mapping vegetation structure with high detail and sampling frequency becomes possible across areas of up to several square kilometers. UAS LiDAR systems have been used to detect individual trees and to measure metrics such as height and stem diameter (Wallace, Lucieer, Watson, & Turner, 2012; Wieser et al., 2017). The ability to detect single trees has been found to increase with higher point densities (Wallace, Lucieer, & Watson, 2014), and Balsi, Esposito, Fallavollita, & Nardinocchi, 2018 could contrast different shapes of horizontal overlapping trees using information from the whole volume of points in the point cloud. Furthermore, Moeslund et al., 2019 analyzed the potential of using airborne LiDAR‐derived metrics, including a biomass measure, to assess the diversity of different organisms (i.e., vascular plants, fungi, lichens, and bryophytes).

The aim of this study was to assess the value of UAS LiDAR for monitoring structural change in a seminatural grassland threatened by encroachment of *C. scoparius*. We developed a semiautomatic workflow to measure structural features of different shrubs and to enable standardized monitoring for estimating spatiotemporal changes of *C scoparius* biomass. Specifically, we addressed the following questions: (a) How accurately can *C. scoparius* be classified in UAS LiDAR point clouds? (b) How precise can the biomass of *C. scoparius* individuals be estimated from point clouds? (c) How does the estimated *C. scoparius* biomass change between two surveys at different seasons?

## 2 MATERIALS AND METHODS

### 2.1 Study area

The study was conducted in a seminatural grassland located within Nationalpark Mols Bjerge, Denmark (56°13′40.0″N; 10°34′30.2″E). The area is situated in a temperate climate zone with a mean annual temperature of 7.5°C and mean annual precipitation of 585 mm (Fick & Hijmans, 2017). The terrain elevation varies between 30 and 60 m above sea level. Graminoids and small, broad‐leaved herbs characteristic for European dry grasslands dominate the vegetation, while single standing trees and different species of shrubs are patchily distributed across the area. *Cytisus scoparius* forms dense stands in parts of the area and a broad‐leaf forest is located toward the northeast. Besides naturally occurring wildlife, the area is grazed by Galloway cattle and Exmoor ponies and thus affected by all year grazing and trampling. The grazers were introduced in 2016 as part of a rewilding experiment for creating a more self‐regulating ecosystem (Svenning et al., 2016).

### 2.2 UAS LiDAR system

**FIGURE 1:**
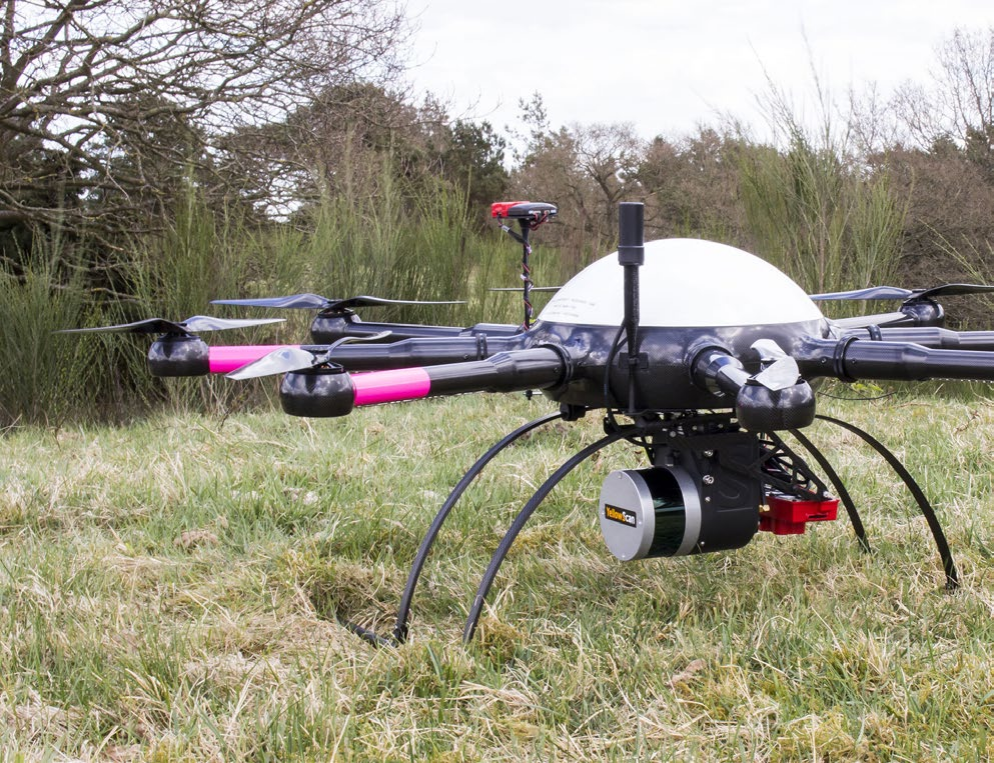
UAS LiDAR system with background of *Cytisus scoparius* shrubs. *Photo credit*: Urs A. Treier

We integrated the Surveyor laser‐scanning system (YellowScan, Montferrier sur Lez, France) on an 8‐rotary wing UAS (MK8‐3500; Mikrokopter, HiSystems GmbH; see Figure 1). The Surveyor is a dual‐return system ranging in the 903 nm wavelength with 360 degrees scanner angle, based on the Velodyne VLP‐16 “Puck” laser scanner with a maximum measurement range of 100 m and a ranging accuracy of 3 cm. The LiDAR sensor payload weighs 1.6 kg. The Surveyor has a global navigation satellite system (GNSS) receiver and inertial measurement unit (IMU) integrated that acts as a rover (Applanix APX15). We utilize a Trimble base–rover postprocessed kinematic (PPK) solution to gain subdecimeter accuracy for the system in XYZ directions (Chaponnière & Allouis, 2016).

### 2.3 Data acquisition

Fieldwork was conducted in October 2017 (leaf‐on) and April 2018 (leaf‐off). Each time the UAS LiDAR system surveyed the area in three separate flight paths planned with the software Kopter‐Tool V2.20b with a flying height of 40 m above ground and a ground speed of 3 m/s. The planned waypoints ensured a standardized flight plan in transects with approximately 15 m between flight paths. We restricted the scan angle to ±55° (roll axis) with respect to the sensor pointing nadir (−90° pitch axis) resulting in an estimated overlap of 65% and thus reducing noise associated with very oblique scan angles.

Between the two flight campaigns, the position of 180 individuals of 12 different shrub/tree taxa (65 of these were *C. scoparius*) was determined with an RTK GNSS receiver on the ground (<2 cm absolute accuracy) for training and validation data. The additional genera measured, included *Juniperus, Rubus, Rosa, Quercus, Betula, Pinus, Sambucus, Crataegus, Prunus, Malus,* and *Calluna*, all collected along random transects with complementary samples of less abundant species outside (Appendix 2).

To validate the estimation of biomass from *C. scoparius*, ten individuals were harvested for dry weight measurements after 2 days of incubation at 60°C. Before cutting the shrubs, they were measured with the RTK GNSS device, resulting in small manual point clouds of 25 points each (Appendix 3).

### 2.4 Processing workflow

**FIGURE 2:**
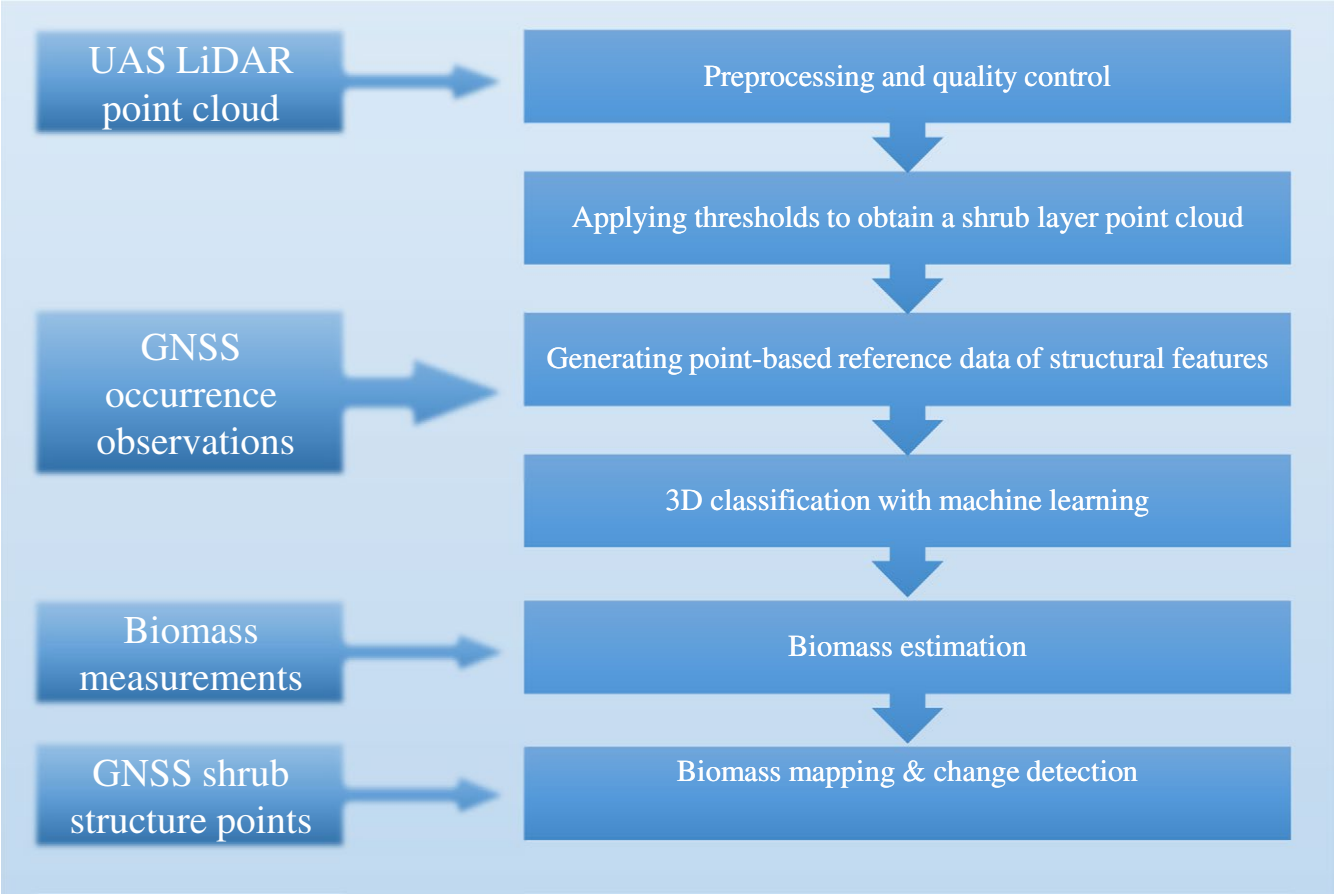
Flowchart of UAS LiDAR processing chain to derive structural information (biomass) from classified shrub species and comparing between two time periods

We developed a workflow for processing ultrahigh‐density point clouds from UAS LiDAR to detect and map structural change in shrubs (Figure 2). Point cloud data processing was performed separately for 2017 and 2018 data with OPALS software v. 2.3.1 specifically developed for handling airborne LiDAR data (Pfeifer, Mandlburger, Otepka, & Karel, 2014).

#### 2.4.1 Preprocessing and quality control of LiDAR data

The position data recorded during the flight by the LiDAR system were postprocessed using PosPac UAV v. 8.2 with data recorded by a Trimble base station to obtain PPK corrected trajectory information and point clouds. All flight lines were carefully considered and selected using a QGIS (QGIS Development Team, 2019) plug‐in provided by YellowScan (YellowScan, 2016) to minimize noisy data from UAS turns. The resulting point clouds were then evaluated with 12 RTK GNSS measurements of fenceposts functioning as control points for the vertical accuracy (RMSE: 3.9 cm in 2017 and 6.1 cm in 2018) and, likewise, five ground markers in 2018 to evaluate horizontal accuracy (RMSE: 1.4 cm).

We performed a relative adjustment of the point cloud to improve the alignment of objects (i.e., vegetation). The procedure was based on fitting overlapping flight strips with a least squares matching technique as described in Ressl, Mandlburger, and Pfeifer (2009). This process aligned the point clouds better relatively by, for example, reducing the variation in ground point height. However, because of the variation in flight strips, the absolute accuracy decreased to a vertical RMSE of 7.6 cm and 9.1 cm for 2017 and 2018, respectively, and a horizontal RMSE in 2018 of 6.5 cm. For further details on preprocessing and quality control, see Appendix 1.

#### 2.4.2 Applying thresholds to obtain a shrub layer point cloud

We coarsely divided the point cloud into height‐based vegetation classes and a ground class to reduce processing time for the later shrub classification. A digital terrain model (DTM) derived from the minimum point height within a 1x1 m moving window was used to calculate normalized height of points (NormZ) (Appendix 2). We then classified the points using thresholds into ground (NormZ < 0.15 m), low vegetation (NormZ < 0.3 m), and high vegetation (NormZ > 3.5 m) leaving a shrub layer point cloud of medium height vegetation (0.3 m < NormZ < 3.5 m). *Calluna vulgaris* fell into the low vegetation layer and therefore was excluded from the further shrub classification procedure.

#### 2.4.3 Generating point‐based reference data of structural features

From the 180 measured GNSS points, we gathered adjacent LiDAR points within a size‐specific area to comprise a reference dataset of 13 classes (11 shrub taxa, fenceposts, and shrub absence points; Appendix 2). Reference data were collected as presence/absence (PA) of shrubs in late 2017 and early 2018, that is, between the UAS flights. Within this period, we would only expect shrubs to disappear, for example, browsing/trampling by animals, which would mean that such individuals will be included as a GNSS record in 2017 but would not be there in 2018. Because of the digital assignment of training/validation data, no points would represent the shrub in 2018, and therefore, no points will be assigned to the given shrub class.

We developed structural features derived from the UAS LiDAR point cloud to represent vegetation morphology with the aim to classify shrub species (Appendix 2). We targeted the variables to represent shrub species on varying levels of scale, ranging from small‐scale leaf characteristics to larger‐scale shrub growth form and shape. The shrub growth form is here interpreted as the general appearance of the shrub and of how branches and leaves are arranged. Hence, it corresponds to specific, although overlapping features, such as structural complexity, density, and light penetration (Popescu, 2011). Specifically, we calculated the variance in height from a fitted normal‐based plane (*Pseudowaveform*) to resemble roughness or structural complexity (Van Aardt et al., 2012). The amount of points (*Point count*) and average distance between points (*Point distance*) were used as a measure of density, while light penetration was represented by including the count of ground points classified by the above mentioned threshold (*Ground points count*) and the average number of returning echoes (*No. of echoes*). Additionally, we calculated a *Rank* feature, where points within the search radius are ranked by lowest to highest point and assigned a corresponding value between 0 and 100. This measure was intended to represent shrub shape and can particularly be useful for recognizing overhanging canopies. Also, characterizing shrub leaf and branch features, the angles between a point and all its neighbors within a search radius were extracted and averaged as the *negative openness* for each point. The *negative openness* refers to a conical view looking downwards, while oppositely the positive would be pointing up. It were originally developed for pixel‐based terrain modeling by taking the mean openness angle from the eight neighboring pixels (or more, depending on the search radius) in each cardinal and intercardinal direction (Yokoyama, Shirasawa, & Pike, 2002). However, all points in any possible direction within the search radius were considered for the 3D point attributes and therefore not necessarily restricted to eight directions.

From the initial set of 17 structural features, we retained seven after testing for autocorrelation and variable importance (Table 1). Variable importance was calculated manually based on the decrease in overall accuracy when leaving one variable out in the classification process (Appendix 2). Variables with high pairwise correlation (*ρ* > .75) and low importance scores were removed from the dataset.

**TABLE 1:** Variables selected for final classification

Variable	Overall importance score	Search radius (m)	Morphological features	Ecological relevance (presumed)	Computation description
Pseudowaveform	0.048	0.5	Shrub growth form	Increases with structural complexity (roughness)	Point height variances from a normal‐fitted plane
Point count	0.013	0.5	Shrub growth form	Increases with surface area and density	Number of points
Ground points count	0.006	0.1	Shrub growth form	Increases with light penetration and decreases with density	Number of points including ground points
Rank	0.002	0.1	Shrub shape and growth form	Highest values at upper surface and lowest at bottom canopies	Relative position in neighborhood. Max value = 100 and min value = 0
Negative openness	0.005	0.1	Shrub shape, leaf orientation, and branch arrangement	Increases with surface gaps	Angular distance between points from a nadir view
Point distance	0.025	0.1	Leaf structure and branch arrangement	Decreases with density	Average linear distance between points
No. of echoes	0.004	0.1	Leaf structure and branch arrangement	Decreases with light penetration	Average number of strongest and last return echoes

#### 2.4.4 Machine learning for 3D classification of *Cytisus scoparius* and other shrub species

For the 3D classification of the shrub point cloud, we utilized the built‐in classification procedure in the OPALS software. It uses the tree‐based decision algorithm (De'ath & Fabricius, 2000) termed recursive partitioning via the *rpart* package for R (Therneau & Atkinson, 1997). The algorithm operates by dividing the dataset for each chosen variable separately, that is, it finds the splitting value for tree branches which results in the purest nodes, that is, most homogenous. A perfectly pure node refers to all observations being assigned the correct label caused by the split value. When the variables could not further increase the node purity, the resulting decision tree was pruned with the complexity parameter set to 0.001 for a final simplified decision tree. We fixed the complexity parameter after a trial process where we lowered the parameter stepwise until the accuracy started to decrease. The aim was to run the classification with the lowest possible complexity parameter.

The classification accuracy was assessed by randomly stratifying 90% of the reference data as training and 10% as testing data. The quality and reliability of accuracy assessments are affected by the reference data input and sampling strategy (Millard & Richardson, 2015). We therefore also assessed a 70% training to 30% validation data split (Appendix 2). Stratification was done among the defined classes. We generated 100 such 90/10 and 70/30 training and validation datasets as input for the OPALS classification algorithm. For each set, classification accuracy was assessed from the resulting confusion matrices using R to calculate overall accuracy and Kappa values, while the class‐wise accuracies were evaluated with precision, recall and the harmonic mean between the two, termed “F1” (Forman, 2003). The 70/30 split resulted in <0.5% decrease in overall accuracy and Kappa coefficient (Appendix 2). To increase the amount of training data used in the final model for extracting biomass metrics, we applied a model validated by the 90/10 split (Appendix 2). From the predictions, we obtained the class probabilities allowing fuzzy classifications, that is, the membership of a class is represented by a probability value between 0 and 1 rather than a Boolean value (true or false) as with traditional hard‐boundary classification(Foody, 1996; Zlinszky & Kania, 2016). Finally, a classification was performed with 100% of the reference data and printed into the full point cloud for visualization purposes.

#### 2.4.5 Assessing variable and model transferability

We used a Wilcoxon signed‐rank test to evaluate the variable signatures for each shrub species in the validation samples from 2017 and 2018 (Appendix 2). Furthermore, the classification procedure was applied to a merged point cloud, consisting of the mixed signatures from 2017 and 2018.

Additionally, we applied a similar classification procedure as described above, but with the variables projected into 2D raster data (Appendix 2). The 2D classification was performed with R statistics 3.5.0 (R Core Team, 2016) allowing us to test alternative classification algorithms.

#### 2.4.6 Biomass estimation and change detection

To estimate the relationship between actual measured biomass and structural information for each of the 10 harvested *C. scoparius* shrubs, we extracted digital volume metrics based on the NormZ variable equivalent to vegetation height (Appendix 3). The NormZ pixel values were computed using mean, max, and range (max–min) values from the points and thereby representing volume metrics by multiplying the height with the pixel area (25 cm^2^). We then used the manually constructed 3D point clouds from the field GNSS measurements to delimit and extract summed volume values for each of the harvested shrubs (Appendix 3).

**FIGURE 3:**
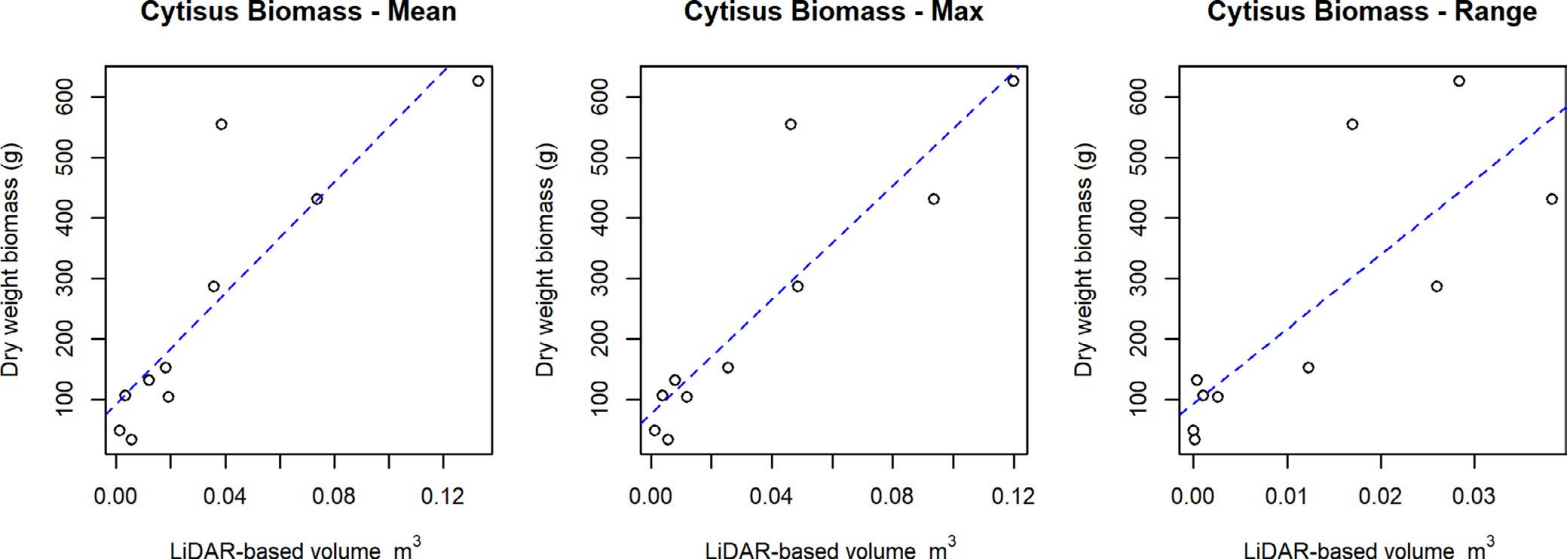
Scatterplot with actual measurements of *Cytisus scoparius* biomass on *Y*‐axis and LiDAR‐based volume on *X*‐axis. Dashed blue lines represents corresponding linear regression models: Biomass ~ Volume for mean, max and range values. ( $R_{\text{adj}}^{2}$ values for the models: mean, max and range values: .72, .77, and .61 with *p*‐values respectively: .0012, .0005, and .0048). Formulas: *Y*(Mean) = 92.49 + 4,575.88*X*; *Y*(Max) = 77.12 + 4,696.16*X*; *Y*(Range) = 92.98 + 12,327.87*X*

The extracted volume metrics were all compared for correlation with nonparametric Spearman's rank correlation using R statistics 3.5.0 (R Core Team, 2016). Furthermore, we developed linear models from the volume metrics to explain biomass from the harvested samples and evaluated them by calculating adjusted R squared values ( $R_{\text{adj}}^{2}$ ) via the lm() function in R. We used $R_{\text{adj}}^{2}$ values from the models to cope with the relatively low sample size of 10 and thereby avoid making too optimistic conclusions. We applied the model coefficients (Figure 3) from the best fit (maximum NormZ) to calculate a biomass estimate for the rasterized variable across a 6.7 ha area and for both datasets (2017 + 2018). During the raster projection, we incorporated class probabilities from the final fuzzy classification models (see accuracy assessment in Appendix 2) to exclude points classified as *C. scoparius* with <60% probability for one set of maximum NormZ values. Likewise, a second set of values was extracted by adjusting this probability threshold more strictly to 80%. At last, the change in biomass was mapped and aggregated from 5 cm resolution to 2.5 m grid cells to emphasize the change.

## 3 RESULTS

### 3.1 Detection of *Cytisus scoparius* in a 3D landscape of points

After georeferencing and noise filtering, our UAS LiDAR system generated 59 million and 53 million points during the 2017 and 2018 flight campaign, respectively, for the 6.7 ha area. For 2017, 12.6% of these points were classified into the shrub layer, and 6.1% in 2018. Likewise, 29.8% and 40.5% of the shrub layer points were classified as *C. scoparius* in 2017 and 2018, respectively (see Appendix 2). The overall accuracy from the 90/10 split classification was 86.9% for 2017 and 95.2% for 2018, while the Kappa coefficient resulted in 83.7% for 2017 and 92.9% for 2018. The 70/30 split resulted in <0.5% decrease in overall accuracy and Kappa coefficient (Appendix 2). Focusing exclusively on the *C. scoparius* class, the F1 measure was 96.2% in 2017 and 98.4 in 2018 (Table 2). The accuracy assessment from the specific model used for obtaining biomass differed with +0.2% in 2017 and +0.5% in 2018 from the mean of the iterative model validation with 90/10 splits (see details in Appendix 2).

**TABLE 2:** Accuracy assessment of the point cloud classifications of *Cytisus scoparius* from autumn 2017 and spring 2018. Classification accuracies are averages from 100 model iterations of randomly selected training/validation data (90/10% split) and with standard deviations. Overall accuracy and Kappa coefficient evaluate the classification of all classes, while the F1 score (Forman, 2003) assesses the performance in predicting the *C. scoparius* class. The last row presents the results from a merged classification including both the 2017 and 2018 point clouds

Time period	Overall accuracy		Kappa coefficient		F1 score—*C. scoparius*	
	Mean	*SD*	Mean	*SD*	Mean	*SD*
Autumn 2017	86.9	0.006	83.7	0.007	96.2	0.005
Spring 2018	95.2	0.004	92.9	0.006	98.4	0.003
Merged	83.2	0.007	78.0	0.010	96.3	0.004

The analogous merged classification showed a decrease in overall accuracy (83.2%) and Kappa coefficient (78.0%), while the *C. scoparius* F1 remained equally high (96.3%) (Table 2). For the more traditional 2D classification approach, the random forest classifier performed in general better with overall accuracies of 45.6% for 2017 and 42.6% for 2018 (see Appendix 2). However, the recursive partitioning classifier did not perform markedly worse (overall accuracy of 47.1% and 38.9% for 2017 and 2018).

**FIGURE 4:**
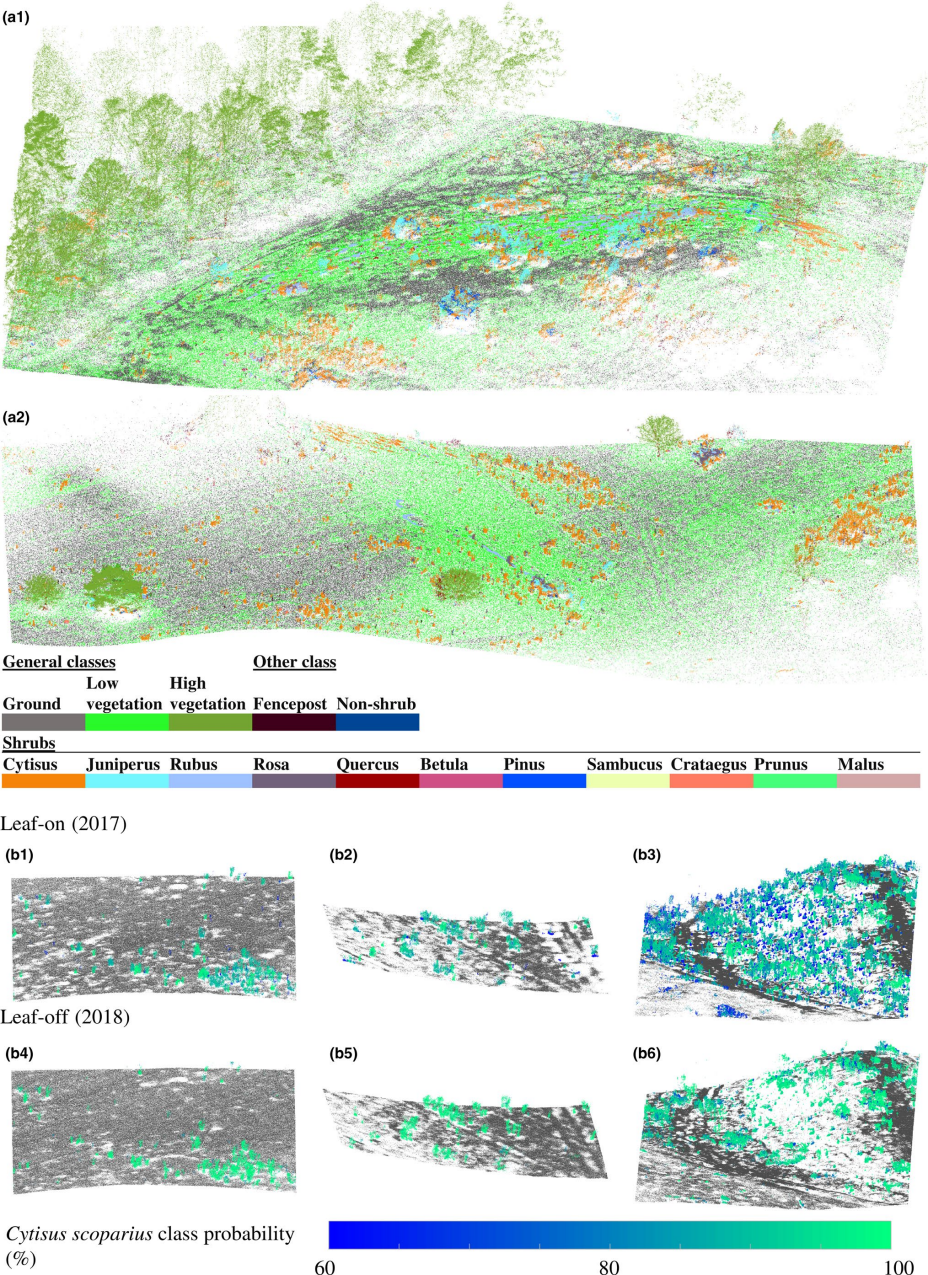
Classification results visualized directly in the 2018 point cloud in a vegetated area (a1) and in more open land (a2). The general classes are showing both, the main vegetation classes obtained from thresholding (Ground, Low vegetation, High vegetation) as well as the classes obtained by the tree‐based classification. The orange colors represent points classified as *Cytisus scoparius*. Lower figures show 3D clips of the 2017 (b1 to b3) and 2018 (b4 to b6) *C. scoparius* point clouds. Points are here color‐coded with the resulting class probabilities ranging from 60% (dark blue) to 100% (light green). The generally classified ground points are colored in grey

The color‐coded point cloud visualizing the resulting shrub classification (Figure 4a) revealed that *C. scoparius* shrubs were detected across the area in various densities, from single individuals to large thickets. When inspecting the classification probabilities in the point cloud (Figure 4b), we recognized a greater certainty to predict *C. scoparius* in the leaf‐off period in 2018 than in the leaf‐on period. Furthermore, it is noticeable that *C. scoparius* shrubs were detected underneath the forest canopy as well (Figure 4a1).

### 3.2 *Cytisus scoparius* biomass estimation

The LiDAR‐derived volume metrics of NormZ (average; maximum; range) correlated well with the biomass measurements of the 10 harvested *C. scoparius* shrubs (Spearman's *ρ* = .87; .88; .88). However, the inclusion of very small shrubs in the harvested samples challenged the LiDAR detection of biomass (Appendix 3). The maximum volume resulted in the best linear fit with $R_{\text{adj}}^{2}$ = .77 (Figure 3). The mean and range metrics performed worse in a linear model with $R_{\text{adj}}^{2}$ = .72 and $R_{\text{adj}}^{2}$ = .60, respectively. The accuracy assessment from the specific model used for obtaining biomass metrics is presented in Appendix 2.

### 3.3 *Cytisus scoparius* biomass change

**FIGURE 5:**
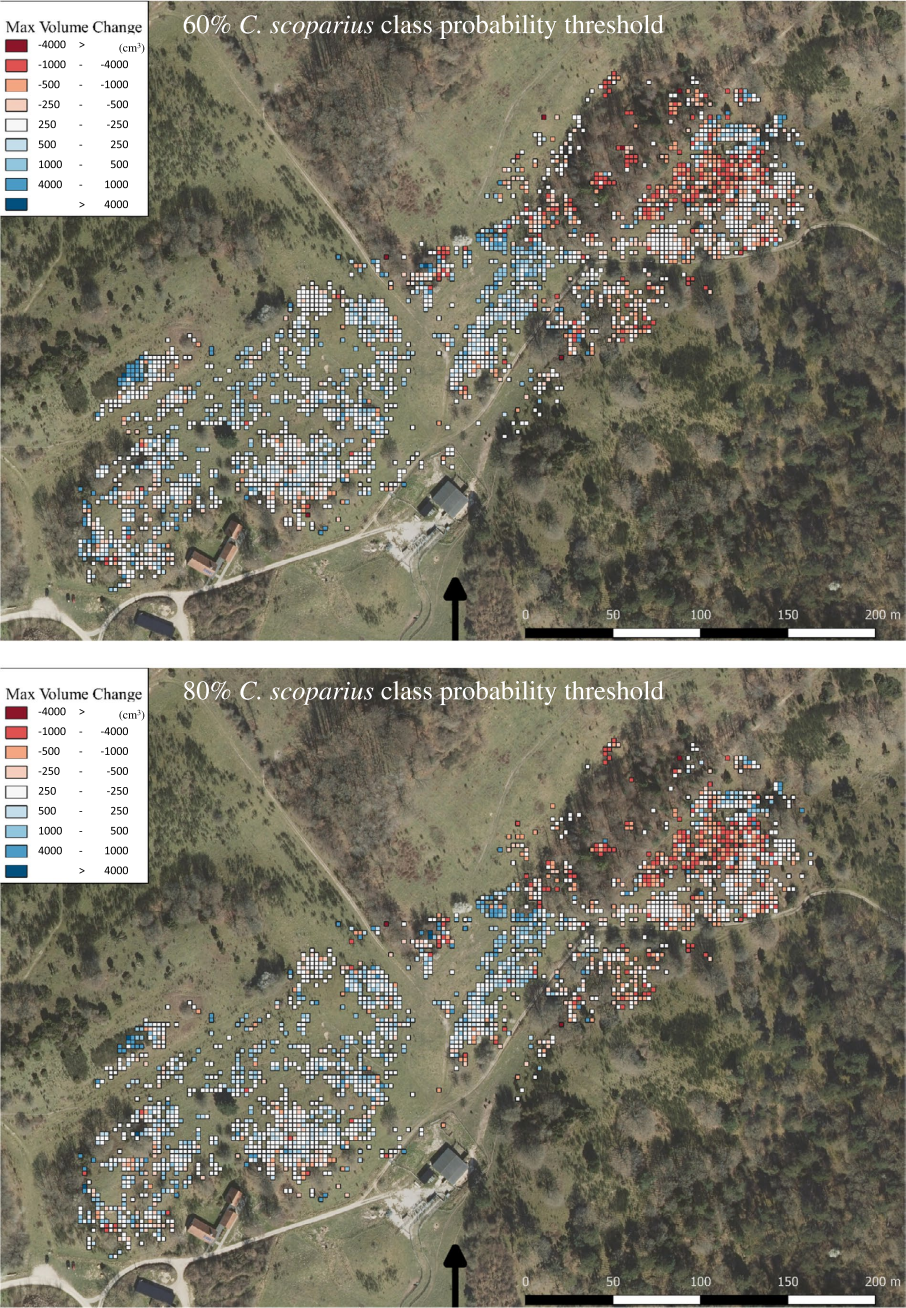
Orthophoto from the study area (Kortforsyningen Danmark, 2019) with overlay of change in maximum *Cytisus scoparius* volume between 2017 and 2018 UAS LiDAR survey. A *C. scoparius* class probability threshold is applied and only allowing points classified with >60% (top) and >80% (bottom) probability to be included for change detection. The change is upscaled from 5 cm to 2.5 m^2^ for visualization. Blue squares represent growth, while red squares show a biomass decrease. White squares indicate no or little change

We extracted the total biomass sum from the points with >60% and >80% probability of being *C. scoparius*. This resulted in 7,500.4 and 5,257.6 kg in 2017 and 2018, respectively, for >60% and, likewise, 5,320.9 and 4,993.0 kg for >80%, in an area of 6.7 ha. For the comparison, we included only the overlapping areas that had an average point distance below 3 cm. On the landscape scale, this resulted in an average biomass decline of *C. scoparius* from autumn 2017 to spring 2018 of 33.4 and 4.9 g/m^2^ for the 60% and 80% probability thresholds, respectively. However, on a local scale the distribution of biomass changes in the area varied but was similar for both thresholds. An upscaled visualization from 5 cm resolution to grid cells of 2.5 m × 2.5 m revealed a pattern of larger decreases in biomass to be identified in especially the northeastern part of the area, while in other parts, we observed no or a slight increase in biomass between the 2 years (Figure 5).

## 4 DISCUSSION

With the sole use of UAS LiDAR‐derived structural information, we identified shrub classes with an overall mean accuracy of more than 86.9% regardless of the year and detected *C. scoparius* with at least 96.2% F1 accuracy in the point cloud (Table 2). Furthermore, using a simple volume metric (NormZ) from the classified *C. scoparius* point cloud, we explained 77% of the variation in actual harvested biomass (Figure 3) and quantified a reduction of 327.8 kg during the winter period from autumn 2017 to spring 2018, assuming the 80% probability threshold to be most accurate.

### 4.1 LiDAR‐derived structural features for shrub classification

Our study demonstrates that ecologically meaningful features can be extracted from UAS LiDAR point clouds which represent the structural appearance (i.e., growth form, shape, and leaf/branch orientation and arrangement) of different shrub species, especially *C. scoparius*. We targeted the variables to represent shrub species on varying levels of scale, ranging from small‐scale leaf characteristics to larger‐scale shrub growth form, density, and shape. For example, *C. scoparius* is characterized as being light competitive (Peterson & Prasad, 1998), hence, reducing the available light beneath the canopy. However, compared with, for example, the compact canopy of *Juniperus communis,* it may allow more light to travel through, especially during leaf‐off periods, eventually resulting in more ground hits of the LiDAR beams.

The variable that explained most of the variation, *Pseudowaveform*, was computed with a relatively large search radius (0.5 m) and therefore represents the variation in the structure of shrub branches and leaves (Table 1). By calculating on this relatively larger scale, we expected the variable to be less affected by differences in leaf abundance between the two flights. From analyzing the shrub signatures, we find *Pseudowaveform* to be stable between the two seasonal states and throughout the classes, except for *Betula* (Appendix 3). Structural complexity in vegetation is often assessed by the variance of derived LiDAR metrics relative to a horizontal plane (Kane et al., 2010; Kukunda et al., 2019). Textural metrics in image analysis can similarly be used to distinguish structurally dissimilar species (Oldeland, Naftal, & Strohbach, 2017), but are limited to detect only 2D surface differences. Here, we utilize information of 3D vegetation structure. The variance for example will be set relative to a normal‐based plane. Measurements of shrub growth form may be difficult to conduct and define in field‐based surveys; hence, there is a focus on retrieving metrics as, for example, diameter at breast height (DBH; Wieser et al., 2017). Nonetheless, the high importance of *Pseudowaveform* for classifying shrub species suggests that shrub growth form is highly relevant for distinguishing species. *Ground points count* and *no. of echoes* express the amount of light penetrating a shrub to represent density. Density is known to be highly variable among species (Van Leeuwen et al., 2011) while light penetration may also reflect habitat characteristics for the species composition (Moeslund et al., 2019). Density was also computed more directly with the amount of LiDAR points within a 50 cm search radius (*point count*), while *point distance* was computed on a smaller scale mainly emphasizing single leaf/branch structure and arrangement. Because of the direct influence from density, it remains challenging to estimate biomass in areas with high species heterogeneity (Wijesingha, Moeckel, Hensgen, & Wachendorf, 2019), and to improve accuracy, it is necessary to separate the species by a classification process. The *negative openness* was used as a metric for differentiating the structural complexity in especially the surface of shrubs. Specifically, the openness variable detects concaveness (Yokoyama et al., 2002) or in this case holes in the vegetation surface. From the shrub signatures, we see that *negative openness* is seasonally affected by a species like *Betula* (Appendix 3) showing lower concaveness in the leaf‐on than leaf‐off period. This can possibly be explained by leaves forming a relatively round and smooth canopy, whereas the branch gaps will be more exposed during leaf‐off period. Finally, to differentiate between *C. scoparius* and low tree species in the shrub point cloud with overhanging canopies, for example, *Malus sp*. or *Prunus cerasifera,* the classification benefitted from the *Rank* variable. Here, lower points from a hanging branch without points underneath would be ranked lower than *C. scoparius* branches, which often will have lower points beneath, from low vegetation or ground. Thus, computing ecologically meaningful variables from 3D point clouds is possible but needs to be based on ecological knowledge and targeted on the focal species or vegetation class.

The performance of the LiDAR‐derived structural variables to detect and distinguish shrub species in this study demonstrates a promising use at the level of detail obtainable with a drone‐based platform (Table 2). In addition to the increased spatial resolution and structural detail, our landscape study of *C. scoparius* differs from Hill et al. (2016) in being independent from observations during flowering periods. However, phenological events can also be considered an important aspect of understanding the development of shrub encroachment. In particular, an increase in temporal resolution would be beneficial for remote sensing studies of vegetation to understand how such variables vary with seasonality. A study by Müllerová et al. (2017) demonstrates an example of this, by recognizing two invasive species from differently scaled images and throughout the season. Hence, using spectral information in combination with LiDAR could extend the monitoring possibilities even further.

### 4.2 Workflow and classification challenges

The use of LiDAR‐derived features poses many challenges and demands a novel way of developing and understanding these 3D measures in an ecological context. Thus, further improvements are possible by implementing ecological or biological knowledge in the computed variables. Depending on the focal species, the LiDAR‐derived features can be adjusted to fit specific morphological characteristics, and a more general approach could be developed to assure transferability for many shrub species. For *C. scoparius,* the change in structural signature between leave‐on and leave‐off is expected to be low due to the small leaves (Appendix 2). However, with the onset of flowering, this might change for some of the variables. Alternatively, for other shrub species it might be important to develop variables that are independent of seasonal states to assure transferability of the classifier. For *C. scoparius,* we found a significant change in the sample distributions in three of the density variables (PCount_ground, PCount, and PDist) which indicates that seasonal variability might be seen in these variables. However, it is the combined signature that is important for the classification process and the distinction among species. When evaluating normalized vegetation height (NormZ) used for biomass estimation, we find no difference between the two flight dates in *C. scoparius*, which is different for *Betula* species. These findings highlight the need for variable adjustments and/or seasonal timing, to obtain reliable biomass estimations for a given target species.

We implemented a workflow to improve the assessment of shrub biomass during encroachment. During the development, we experienced several challenges which could affect the results and need careful consideration and evaluation during implementation: (a) misalignment reduce spatial accuracy, (b) thresholding of major vegetation classes, for example, trees versus low vegetation, (c) sampling of training data, and (d) selection and development of targeted structural features. Below we outline these challenges and provide perspectives on future directions.

Because of observed misalignments in the combined point clouds, resulting from different laser beams, flight strips, and uncertainties in absolute GNSS positioning, we rectified the relative accuracy in the datasets, which negatively influence the absolute accuracy, that is, absolute geographic position of LiDAR points. However, the decrease in precision of XYZ‐coordinates was relatively small (RMSE: vertical <1.4 cm and horizontal <5.1 cm). When considering the scale of which we recognize *C. scoparius,* we believe that a high relative accuracy among points is more important for describing the structural features of a shrub properly. However, in monitoring programs, fixed features (i.e., ground control points) allowing to align multiyear data would be advisable.

Threshold approaches to filter data noise as well as for doing a first general classification are essential for effective data processing. However, finding meaningful thresholds in heterogeneous plant communities with highly variable structures is difficult and hard to generalize across a larger area. Therefore, deciding on exact thresholding values often is a trade‐off issue, by either including an amount of noise or excluding useful data. In our study, we emphasized to include as much data as possible, accepting some levels of uncertainty. Still, with broadly defined threshold values, points representing a target species may disappear in these first steps of filtering and classifying. For example, we attempted to include training data from the small‐sized shrub *Calluna vulgaris*; however, these points were set aside in the general class “Low vegetation” and thus not included in the tree‐based classification. Reducing the dataset is crucial for optimal processing speed and point cloud visualization but choosing general classes and thresholds must be evaluated and depends on the target species. If targeted, the *C. vulgaris* shrubs could most likely be identified by running the classification procedure on the low vegetation points and could perhaps be implemented in a future study including herbaceous vegetation.

Generally, one should aim for balanced training/validation data (Millard & Richardson, 2015). However, this can be challenging when working in natural areas with a patchy and heterogeneous distribution of vegetation. First, a random sampling strategy to collect data objectively is desired, a strategy we pursued with the transect approach. However, this method may only cover the more dominant species and is not enough to catch the full variation of shrub species in an area. Second, it might be impossible to equally sample rare species occurring only in few places, even if all individuals are sampled. We therefore included datapoints outside our transects from less abundant shrub species, as it would not present the full picture of the area if ignoring those. When covering extents of several hectares, we are aware that some species are possibly missing in the training data, but that does not mean that they do not exist in the point cloud.

It is important to design point‐based attributes to emphasize structural features, corresponding to the species or vegetation type of interest. The described workflow is semiautomatic, given a comprehensive input dataset of UAS LiDAR point clouds, training data for classification, and ground truth data for developing the biomass model. Depending on the study focus and target species, it is possible to design point‐based attributes that emphasize structural features, corresponding to a species or vegetation type of interest. For example, different search radii are required when studying differences in trees or herbs matching the level of scale. Likewise, attributes can be modified to detect specific structural features of species or individuals, for example, deformity caused by pathogens. The structural uniqueness, as defined by growth form, density, roughness, and light penetration seen in different shrub species may be less influenced by seasonality and physiological stress factors (i.e., water and nutrient availability) than spectral signatures. However, when attempting to distinguish structural similar species including spectral information in the classification procedure might help (Zlinszky et al., 2014).

### 4.3 Spatial patterns of *C. scoparius* biomass change

Depending on the probability threshold for detecting a *C. scoparius* shrub, we estimated a decrease in biomass change of 4.9–33.4 g/m^2^ between the two mapping dates. Nevertheless, we found a consistent spatial pattern of biomass change for both thresholds, indicating a large decline in the northeastern part of the area and no or a slight increase in the southwestern part (Figure 5). There might be several reasons for this observed spatial pattern:

First, the slight increase in biomass could result from late autumn or early spring growth. The shrubs are not expected to grow during a Danish winter season; however, the timing of flight campaigns may still include some late autumn or early spring growth. In addition, *C. scoparius* branches contain chlorophyll, which might be able to induce growth in warmer periods and favor early growth in spring. The reason why biomass increase was mainly observed toward the southwest of the study area could be low inter‐ and intraspecific competition or variation in microclimatic conditions. A freestanding individual without neighbors is more likely to grow and expand due to lack of competition when the conditions are suitable. In contrast, competing neighbors may supress growth of *C. scoparius* individuals in densely populated surroundings. The possible intraspecific interaction has previously been raised in the literature (Paynter, Fowler, Memmott, & Sheppard, 1998), and in accordance with this, the observed spatial pattern in biomass changes suggests that *C. scoparius* in the open land (midwestern part) tended to slightly increase, while the denser stands toward the northeast of the study area showed a decline in biomass (Figure 5).

Second, the reasons for a biomass decline could be either leaf fall (Peterson & Prasad, 1998), which due to the summed 2.5 m grids, is expected to be larger in denser stands, or external factors, such as grazing or frost. Galloway cattle and Exmoor ponies graze the area as part of a rewilding initiative, and no additional food sources are supplied to the animals. Hence, at wintertime when the green vegetation becomes sparse, the animals might feed on hardy shrubs like *C. scoparius*, or cause damage to shrubs by trampling. Therefore, one alternative hypothesis explaining the spatial pattern of *C. scoparius* biomass change is that the animals are favoring the northeastern part of the study area. This area is substantially more forested, and the grazers might benefit from shelter provided by the trees during harsh winter conditions. Alternatively, abiotic conditions linked to topographic features, such as light availability and freezing temperatures during winter might limit plant growth and harsh winters would potentially cause *C. scoparius* to die (Peterson & Prasad, 1998). Extracting such topographic variables describing light availability and protection against freezing could provide predictors for the observed change in biomass. The availability of terrain topography data from the UAS LiDAR point cloud would facilitate such a follow‐up study at very high resolution.

While the observed pattern in biomass change is independent of the probability thresholds for *C. scoparius* detection, the absolute biomass change might be overestimated. When comparing *C. scoparius* class probabilities in three areas with varying population densities (Figure 4b), there are more *C. scoparius* points in 2017 than in 2018, especially in areas with dense stands. In the leaf‐on period (2017), a larger number of forbs, tall grasses, and shrubs have probably been included in the training data for *C. scoparius*, as they grow within and around shrubs. This probably also explains why in 2017 more *C. scoparius* points have been classified with lower probabilities than in 2018 (Figure 4b and Appendix 2). More LiDAR points included from entangled vegetation can lead to a larger volume estimate per individual and probably cause slight overestimation of *C. scoparius* biomass in 2017.

### 4.4 Relevance for nature management

The spatial variation in *C. scoparius* biomass change indicates that shrub dynamics differ in the subareas of the mapped area, possibly due to varying importance of ecological drivers, also yet to be studied (Figure 5). Our findings highlight that detection of change in shrub density and biomass with high resolution is important when assessing shrub encroachment in monitoring programs for nature management. Traditionally, remote sensing studies have classified vegetation with a two‐dimensional approach from spectral information or rasterized LiDAR information. These raster‐based methods are efficient in identifying cover or presence of certain grassland species and vegetation types (Hellesen & Matikainen, 2013; Zlinszky et al., 2014). However, to quantify biomass changes and to fully understand the effect of shrub encroachment on plant diversity more comprehensive knowledge is needed. While LiDAR observations from a manned aircraft were applied to map coarse‐scaled shrub encroachment characteristics from a single species (Sankey et al., 2013), we are now able to separate species or genera directly in the point cloud and to detect fine‐scale biomass dynamics from a target species by utilizing a drone platform. With the use of the established workflow based on a UAS LiDAR system, we provide a new approach for monitoring shrub species dynamics. In this study, *C. scoparius* is covering the spatial extent corresponding to management operations, but also capturing the local‐scale information needed for detecting change and its spatial variation. Again, the benefits of using LiDAR are the vegetation penetration ability (Lefsky et al., 2002) and our findings suggest that LiDAR‐derived point clouds are of such a quality, that detecting species of interest even beneath a covering canopy is achievable, as, for example, forest understory species. This will make it possible to monitor and guide management programs for noxious invaders such as *Rhododendron ponticum* (Sanders, 2017) or rare species such as *Allium tricoccum* beneficial for indicating favored nature or ecological conditions (Leduc & Knudby, 2018). In a study by Chance et al. (2016), LiDAR is utilized to map the distribution of two invasive shrub species in an urban environment with reported lower accuracies for shrubs beneath closed canopies. The importance of mapping understory vegetation is further highlighted in a review by Hernandez‐Santin, Rudge, Bartolo, & Erskine, 2019, and the findings from our study should be relevant and encouraging to further studies in this direction.

Shrub encroachment is influenced by several factors that makes it a complex issue for management and conservation at landscapes and regional scales (Cao et al., 2018). The presented workflow provides a potential improvement on several aspects of nature monitoring. By the semiautomatic processing and the use of a UAS‐based platform, the temporal resolution is no longer limited to snapshot observations, for example, once a year or less. Hence, field operations can be carried out on multiple times through a season, with the inclusion of beneficial seasonal states of vegetation. However, we also demonstrate that UAS LiDAR data allow for comparable measures of shrub encroachment even in different seasonal states and hereby displaying the potential multipurpose use from this type of data. Our comparison with a similar 2D remote sensing classification approach (Appendix 2) highlights the benefits from using three‐dimensional information. Hence, we increase the spatial dimensionality from traditional 2D mapping studies by recognizing shrub taxa in three‐dimensional space contributing to reliable biomass estimation which is much needed for deeper understanding of shrub encroachment dynamics. With the fine‐scale information about biomass, it will be easier to detect and manage dense stands of *C. scoparius* or other shrub species during encroachment with potential negative effects on plant diversity (Kesting et al., 2015).

## 5 CONCLUSION

Our study presents a novel method for assessing shrub dynamics in 3D, based on the arrangement and orientation of points in space. We demonstrate an efficient way of determining specific structural features for classifying shrub species. Seven different point‐based structural variables were developed based on ecological knowledge to distinguish *C. scoparius* from other vegetation. Using a tree‐based classification procedure, we identified 11 different shrub species with an overall accuracy of 86.9% and 95.2% in two independent point clouds acquired in 2017 and 2018, respectively. Derived from the point cloud derived height‐based maximum volume of *C. scoparius* shrubs, we established a linear model to explain 77.4% of the variation seen in actual measurements biomass from 10 harvested and weighed individuals. Projected biomass in *C. scoparius* with an 80% class probability declined by 4.8 g/m^2^ between 2017 and 2018. However, we found substantial spatial variation in biomass change with a patchy distribution of *C. scoparius* decrease and or growth. These findings suggest that the combined information from small‐ to large‐scale observations is necessary to fully understand shrub encroachment and highlights the potential application of this method for fine‐scale management of *C. scoparius* populations on a landscape scale. Moreover, our study encourages more studies investigating the causes of *C. scoparius* biomass change variation and its effect on plant diversity.

Our study contributes with new and advancing methods within the field of remote sensing, ecology and nature management by demonstrating an application for ecological monitoring. Natural systems in Denmark, including grasslands, are monitored by the Danish national monitoring program (Svendsen, Bijl, Boutrup, & Norup, 2005). The promising results of detecting change in biomass related to encroachment of *C. scoparius* may inspire improving some of the traditional field‐based measurements toward a more objective evaluation on the state of natural systems. The presented work potentially improves monitoring programs in nature management as it allows to quantify biomass change timely and at a fine spatial scale informing managers to implement management strategies that help sustaining biodiversity during shrub encroachment.

## CONFLICTS OF INTEREST

The authors declare no conflict of interest.

## AUTHOR CONTRIBUTIONS

**Bjarke Madsen:** Conceptualization (lead); data curation (lead); formal analysis (lead); investigation (lead); methodology (lead); project administration (lead); resources (lead); visualization (lead); writing – original draft (lead); writing – review and editing (lead). **Urs A. Treier:** Conceptualization (lead); data curation (equal); formal analysis (equal); investigation (equal); methodology (equal); project administration (equal); resources (equal); supervision (equal); validation (supporting); visualization (supporting); writing – original draft (supporting); writing – review and editing (supporting). **András Zlinszky:** Formal analysis (supporting); investigation (supporting); methodology (supporting); software (supporting); validation (supporting); writing – original draft (supporting); writing – review and editing (supporting). **Arko Lucieer:** Investigation (supporting); supervision (supporting); validation (supporting); writing – original draft (supporting); writing – review and editing (supporting). **Signe Normand:** Conceptualization (equal); formal analysis (equal); funding acquisition (lead); investigation (equal); methodology (equal); project administration (equal); supervision (lead); validation (equal); writing – original draft (supporting); writing‐review and editing (supporting).

## Figures and Tables

**FIGURE A1.1:**
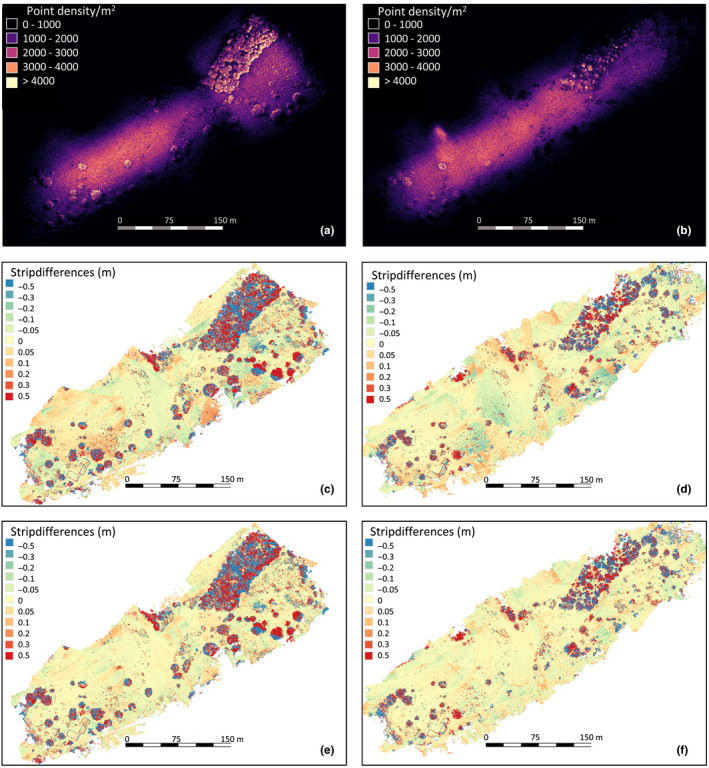
Top figures show maps of point density from Mols 2017 flight (a) and Mols 2018 flight (b). The middle figures presents the strip‐differences calculated from Mols 2017 (c) and 2018 (d) before the relative georeferencing procedure and bottom after the relative georeferencing

**FIGURE A1.2:**
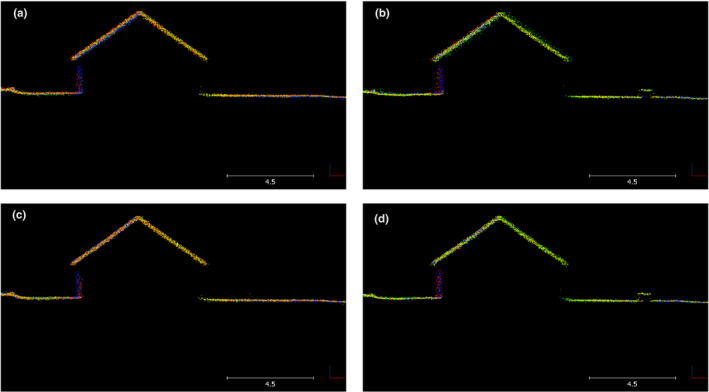
Cross‐sections from Mols 17 flight (a and c) and Mols 18 flight (b and d) before (top) and after relative georeferencing (bottom). Points are colored by the Point ID from each flight strip

**FIGURE A1.3:**
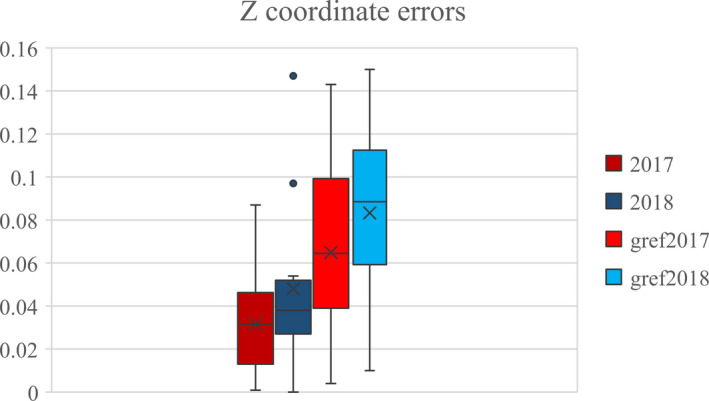
Boxplot showing Z coordinate deviation measurements from 2017 and 2019 surveys before and after relative point cloud adjustment (gref). Outliers are presented as circles, while median and mean values are marked with a line and cross, respectively

**FIGURE A1.4:**
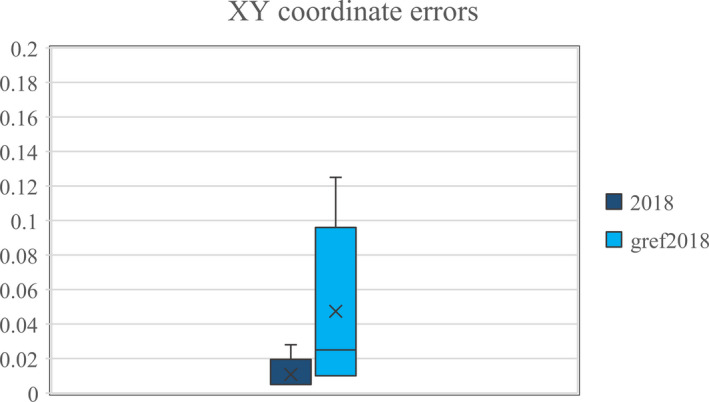
Boxplot showing XY coordinate deviation measurements from 2018 surveys before and after relative point cloud adjustment (gref). Median and mean values are marked with a line and cross respectively

**FIGURE A1.5:**
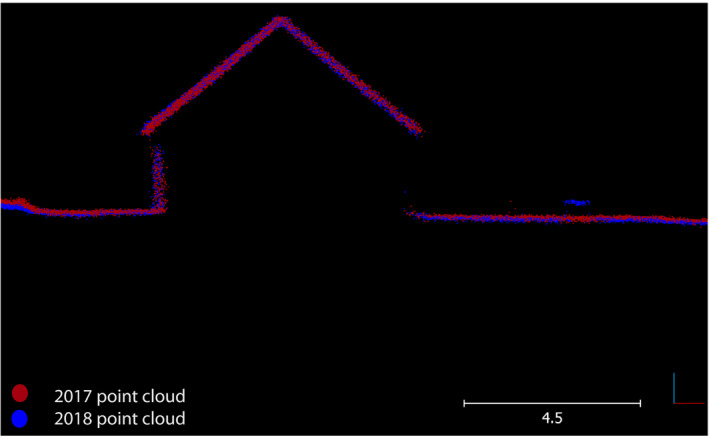
Cross‐section illustrating the point cloud accuracy between flights from Mols 2017 (Red) and 2018 (Blue). Bars in the lower right show the scale bar in white (values in meters), while the point cloud orientation is presented as blue (Z‐axis) and red (X‐axis)

**FIGURE A2.1:**
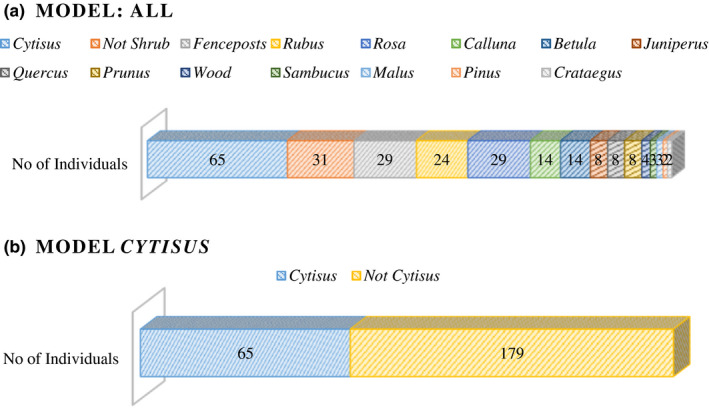
Training and validation data for classification with separate classes of all shrub species (a) and where only the class of interest is kept and all other species was pooled into one class (b). The number of individuals measured within each class is printed on the figure

**FIGURE A2.2:**
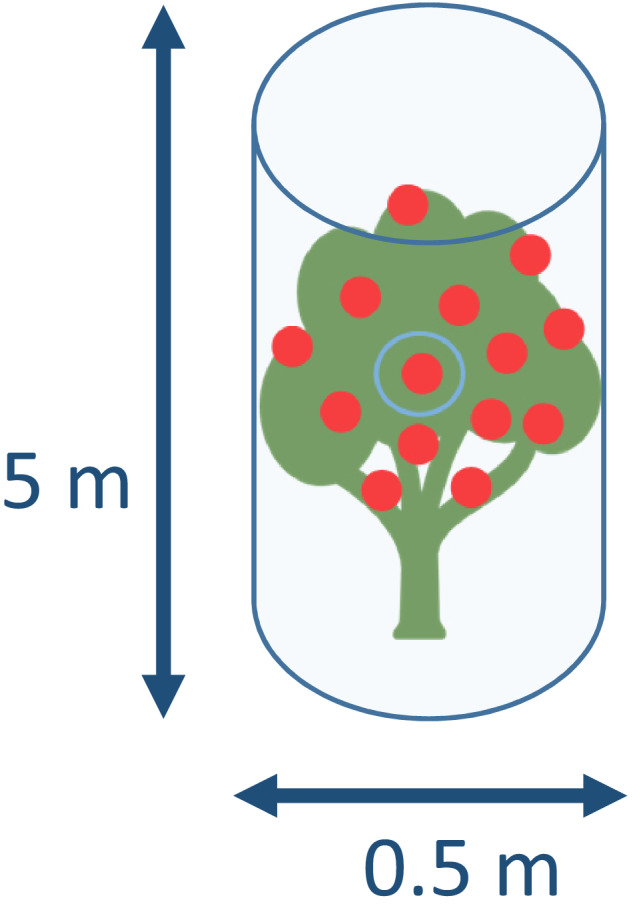
Sketch of averaging cylinder used for homogenizing point cloud attributes to characterize a whole shrub or larger parts of it. Central point indicated by blue circle is assigned the average attribute value based on all the points contained in the cylinder

**FIGURE A2.3:**
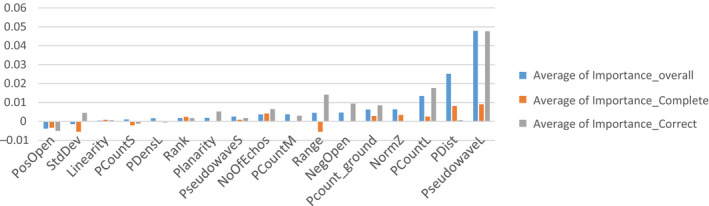
Average values of variable importance from four different models, distinguishing the overall importance (blue), completeness (orange), and correctness (grey)

**TABLE A2.2:**
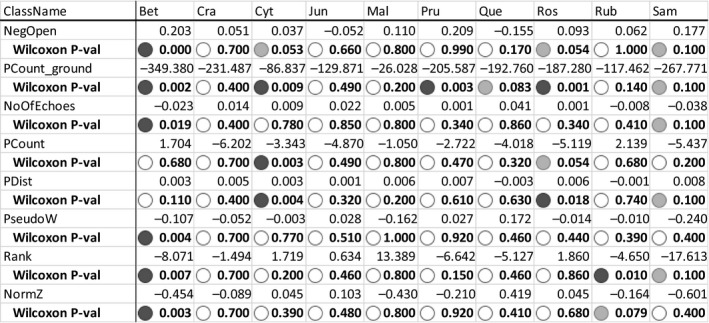
Differences in shrub signatures between 2018 and 2017 flights for each variable and corresponding Wilcoxon *p*‐values comparing the samples. *p*‐Value significance codes are illustrated by circles: *p*‐val ≤.05 (black); *p*‐val ≤ .1 (grey); *p*‐val >.1 (white)

**FIGURE A2.4:**
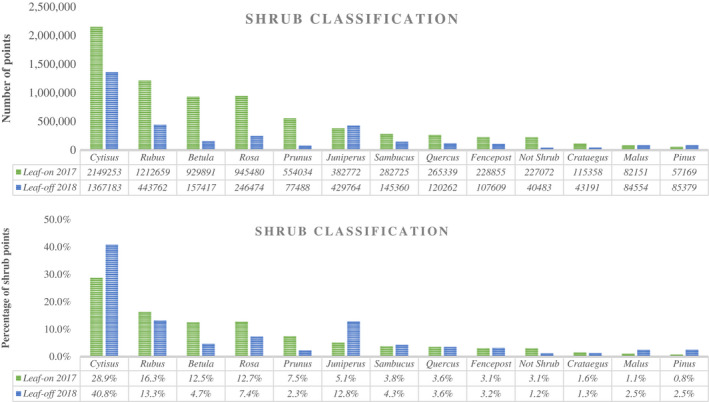
Distribution of classified shrub points from the 2017 survey (green) and 2018 survey (blue) in numbers (top) and percentage of total shrub points (bottom)

**FIGURE A2.5:**
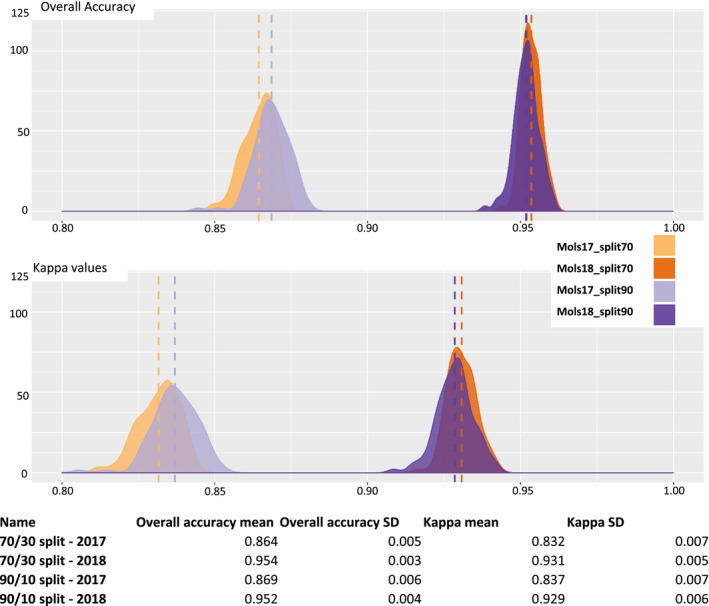
Density plots showing the resulting accuracy distribution from 100 permutations of the classification models, including both the 2017 and 2018 flights. The accuracy metrics used for the general classification are overall accuracy and Kappa coefficient, each shown with the subsets selected by a 90/10 or 70/30 split of the training/validation data. Below the density plots, corresponding tables highlight the calculated mean and standard deviation (SD) for each distribution

**FIGURE A2.6:**
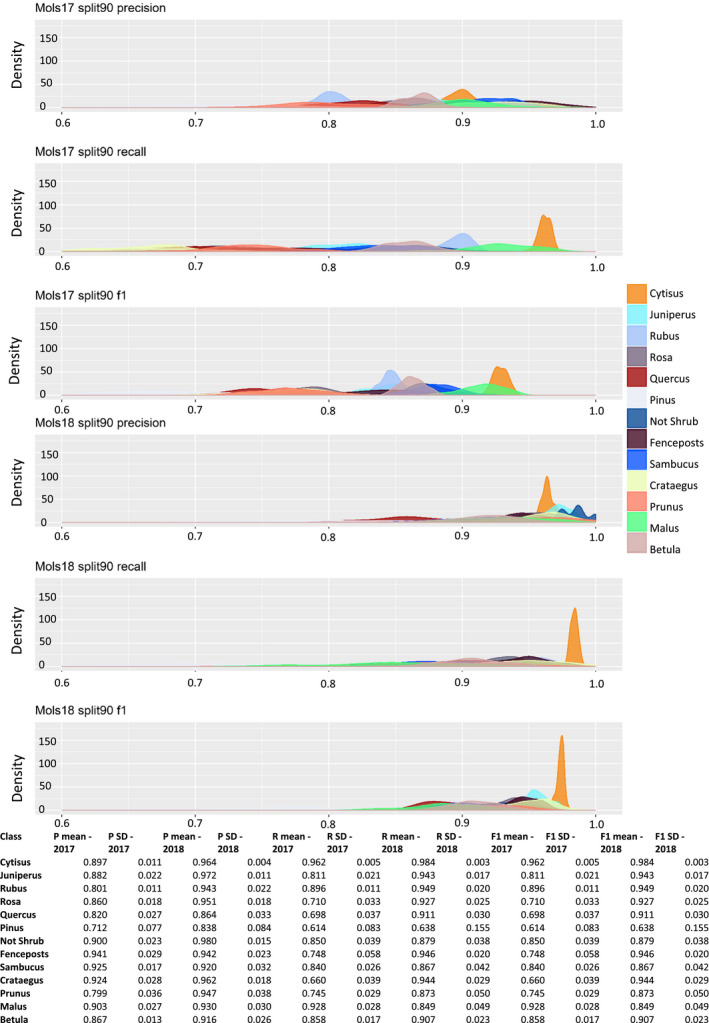
Density plots showing the resulting class‐wise accuracy distribution from 100 permutations of the classification models for the 2017 (Mols17) and 2018 flights (Mols18). The class‐wise accuracies are evaluated on the 90/10 split with the F1 value, based on the precision (P) and recall (R) values (Forman, 2003). Below the density plots, corresponding tables highlight the calculated mean and standard deviation (SD) for each distribution

**FIGURE A3.1:**
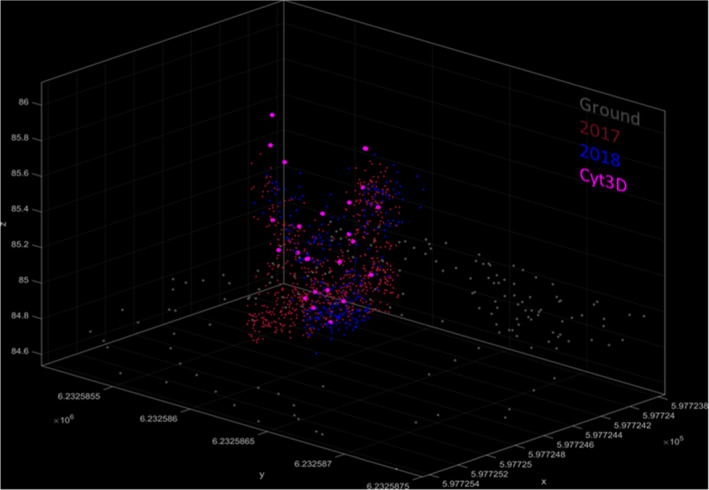
A *Cytisus scoparius* individual represented by LiDAR derived point clouds from 2017 (Red) and 2018 (Blue) and by the manually constructed point cloud Cyt3D (Pink). As a reference, the terrain points are visualized as grey points

**FIGURE A3.2:**
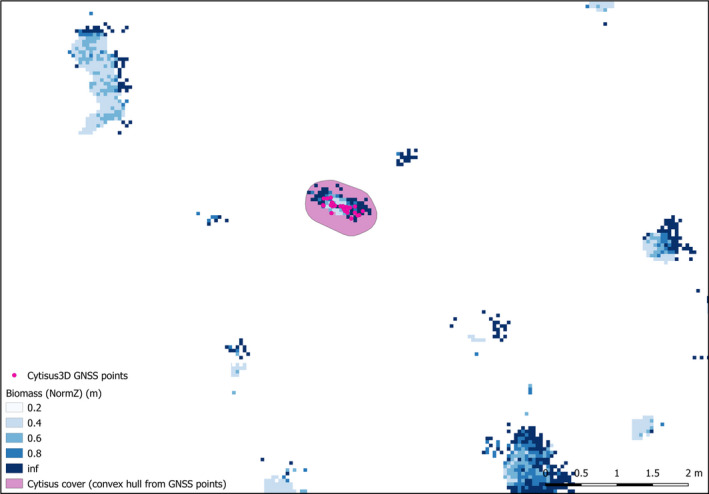
Raster image of normalized height values (blue shades) from *C. scoparius* point cloud in 5 cm pixels. Pink dots represents 25 manually constructed points (Cyt3D), which were used to delimit the convex hull area of an individual shrub with 25 cm buffer (pink area)

## Data Availability

The data used in this study are available through the Dryad Data Repository: https://doi.org/10.5061/dryad.547d7wm55.

## Appendix 1

### LiDAR point cloud processing and quality control

This document describes the procedure UAS LiDAR point cloud processing, quality control, and relative position adjustment. Point clouds have been collected from UAS LiDAR flights in Mols Bjerge, Denmark, 6 October 2017 and 23 April 2018.

#### Pre‐processing

Prior to these analyses, the point clouds had been post‐processed and geo‐referenced using the software POSPac UAV (v8.2, Dec 2017, Applanix, Richmond Hill, Ontario, Canada). From three flights in each year, 32 strips in 2017 and 43 in 2018 were selected for further analysis using the Yellowscan QGIS plugin (YellowScan, 2016). This tool basically determines the GPS‐time used as delimiter for the flight strip data to be further processed. These strips were afterwards re‐adjusted, to make sure that data gathered while the UAS was turning were excluded. At last, data were delimited to a scan angle of ±55 degrees minimizing the amount of noisy points in the dataset.

#### Quality control

Quality was assessed by creating point density maps to evaluate the coverage throughout the area of interest (Figure A1.1a,b). Likewise, the dislocation differences between the overlapping flight strips were calculated and visualized on raster maps (Figure A1.1c–f) as well as in cross‐sections of the point cloud (Figure A1.2). Thickness of the ground points layer was evaluated by measuring a cross section in a place with a minimum of vegetation (i.e., ground thickness should be low) using the point measurement tool in CloudCompare (v2.10, GPL software, 2019, retrieved from http://www.cloudcompare.org/).

#### Relative adjustment of point cloud

It is possible to reduce the differences between overlapping flight strips (i.e., relative accuracy) caused by system inaccuracies (e.g., GNSS and IMU precision) by applying a least squares matching (LSM) technique to each overlapping strip pair (Ressl et al., 2009). These errors are visible as rectangular artifacts in Figure A1.1c–f and as differently colored layers in Figure A1.2, each caused by disjunct flight strips. The adjusting procedure is performed with the GeoRefApprox module in OPALS (v2.3.1, 2018, TU Vienna, Austria, http://geo.tuwien.ac.at/opals/html/ModuleGeorefApprox.html) by keeping one strip as a reference and adjusting the others relative to this reference strip. The reference strip was chosen as the closest to the median position of all strips, which is the standard setting. After the relative geo‐referencing, the quality control was performed once more and visualized for comparison (Figures A1.1 and A1.2).

#### Outliers

Outliers in the point cloud, that is, erroneous records of LiDAR returns, will affect further processing and the quality of point classification. Hence, such outliers have been deleted by attributing each point with the number of points within a search radius of 1 m, that is, a sphere with a diameter of 2 m around the given point and then applying a two‐step procedure. First, we deleted all points that had no neighbors within the 1 m search radius, which corresponds to single noise points. Second, as noise can appear in clumps we also deleted all points with average number of neighbors <3 from points within a search radius of 3 m, that is, a sphere with a diameter of 6 m around a given point.

#### Absolute precision

We measured 12 fencepost‐tops with a differential GNSS system (Trimble Real‐time Kinematic) with centimeter‐level precision. The measured GNSS points were compared to the closest point recognized as a fencepost in the point clouds. The deviation in the Z coordinate could then be measured for each fencepost, which could be distinguished in the point clouds. We found an average deviation of 3.2 cm in the 2017 data and 4.8 cm in 2018, which is within the accuracy of 5 cm given by the technical specifications of the system (YellowScan Surveyor Data Sheet, https://www.yellowscan‐lidar.com/products/surveyor/). However, the accuracy of the absolute position of these control points decreased with deviations of 6.5 cm and 8.3 cm, respectively, after the relative geo‐referencing of flight strips, that is, relative adjustment of the point clouds (Figure A1.3).

In addition, we measured the systems XY precision in the 2018 survey by using five ground control markers. Here, we found the deviation of 1.1 cm to increase to 4.7 cm after the relative adjustment (Figure A1.4).

Using these measurements, we calculated the root mean square error (RMSE) and mean absolute difference (MAD), which are presented in Table A1.1.

**TABLE A1.1:** Calculated root mean square error (RMSE) and mean absolute difference (MAD) from vertical deviation measurements in CloudCompare before and after relative adjustment (*_adj)

	Vertical deviation				Horizontal deviation	
	2017	2018	2017_adj	2018_adj	2018	2018_adj
RMSE	0.039484	0.060631	0.076016	0.091212	0.014	0.064713
MAD	0.018583	0.025667	0.03175	0.030532	0.00696	0.03888

At last, we overlaid the point clouds from each year to visualize the consistency of the two surveys (Figure A1.5). It is noticeable that the red points (2017) seem a bit higher located for ground points most probably a vegetation effect in the leaf‐on period. Overall, the red and blue points are still in seemingly good aligned/intermixed.

#### Concluding remarks

Our findings indicate that the UAS LiDAR system used for this study performed with an absolute position error below 10 cm in both, vertical and horizontal directions. The absolute accuracy is lower than reported in the technical specification for the LiDAR system; however, the assessment is based on the position of fencepost which has been measured with a GNSS RTK system providing ≤2 cm precision. Furthermore, we applied a flight strip adjustment procedure that increases the relative accuracy of the point clouds on the expense of slightly shifting the point clouds in absolute geographic space. Precise alignment of the point clouds was, however, considered most important for the overall purpose of classifying shrubs.

## Appendix 2

### Classification procedure

This document provides details on the entire classification process from preparing and selecting attributes to applying validation data for a tree‐based classification model. The data were extracted from point clouds collected from UAS LiDAR flights in Mols Bjerge, Denmark, 6 October 2017 and 23 April 2018.

A training dataset and feature variables to characterize the targeted classes are essential inputs in supervised machine learning classification approaches (Waldhauser et al., 2014). The classification workflow targets the recognition of *Cytisus scoparius* shrubs in a highly heterogenous and vegetated area of semi‐natural grassland. The workflow in this appendix is described in a three‐step procedure of (a) preparing and training the model, (b) computing and evaluating input attributes, and (c) final classification results.

#### Preparing and training the model

Prior to the machine learning process, the full point cloud was divided into ground, low vegetation, high vegetation, and a shrub layer of points. The thresholding procedure was based on the normalized point height (NormZ), which is calculated by subtracting a 1 × 1 m digital terrain model (DTM) derived from the lowest points from the height of each point.

All points with NormZ < 0.15 m were assigned as ground temporarily. Before concluding on the final ground classification, we transferred points in low densities (<10 pts in spheres with 0.25 m radius) back to non‐ground, to adjust for wrongly classified points in less penetrable 1 × 1 m cells. Second, we calculated a mean of NormZ within cylinders corresponding to vegetation sizes (low vegetation dimensions: 0.1 m radius and 0.3 m height; high vegetation dimensions: 0.25 m radius and 5 m height). We then thresholded the points into low vegetation (NormZ < 0.3 m) and high vegetation (NormZ > 3.5 m) leaving a shrub layer point cloud of medium height vegetation (0.3 m < NormZ < 3.5 m).

##### Training a tree‐based model

Two separate sets of reference data were collected in between the two flight surveys. They consist of single differential GNSS measurements of different objects in the area. In the period between reference data and LiDAR flights no major change to, for example, shrubs are expected and following the described method below for generation of validation points, it should be safe to use the two datasets individually and together.

We measured in total 180 shrub individuals from 12 different shrub/tree taxa with an RTK GNSS device, to create the dataset for training and validation. The reference data gathering was performed in random transects throughout the area and spaced approximately 50–100 m. Along each transect every 100 m, the nearest shrub of each species in the area was sampled, where each shrub was measured as close to the central stem as possible. Additionally, extra samples outside the transect paths were made to complement the training data for less abundant species. The training dataset was used in two different divisions to evaluate possible influence of an unbalanced training dataset. The full model “ALL” contains all measured points in their original separate classes, and the number of individuals within each class is presented in Figure A2.1. Whereas Model “*CYTISUS”* shows the balance of training data when only the focus class *C. scoparius* was kept by itself, while all other classes were me.

##### Generating training and validation points

Because Opals modules for attribute assignments are not always working with shapefiles the reference data needed to be pre‐processed for implementation in an Opals data manager file (ODM).

Buffers of 25, 35, and 50 cm were created around the reference datapoints in QGIS and selected appropriately to cover a single object of each class. The 50 cm buffer was used for all shrub classes except *Calluna vulgaris,* which would be decreased to 35 cm buffer radius along with the Not Shrub class. The fenceposts themselves are varying in radius between 10 and 20 cm, and as they would not always be completely straight, the 25 cm buffer was used here.

In QGIS, the buffered reference points were rasterized to 5 × 5 m GeoTIFF's for each class displaying the reference cells with 1 and the rest of the flight area with 0.

Having the reference information in raster files allows it to be stored as attributes in the working ODM with OpalsAddinfo. Afterwards, all points overlapping in 2D with the raster cells equal to 1 could be assigned as validation points in the given class. To ensure no classes overlapped in the 2D buffer area, the ground, low vegetation, and high vegetation classes were assigned separately.

This method of generating validation points in a point cloud ensures that no false points are introduced in the training model as the actual points are measured with the LiDAR system itself, and the reference data used as a guide for assigning the points.

#### Computing and evaluating input attributes

##### Input attributes

The tree‐based classification procedure available in the OPALS software package is applied on a point‐based level, meaning that each point is classified independently from other points. However, before the classification process is initiated, each input attribute was averaged within a cylinder with radius 0.25 m and height ±2.5 m from every point (see Figure A2.2). In this way, the “salt and pepper effect,” known also from image classification (Blaschke, Lang, Lorup, Strobl, & Zeil, 2000), is avoided and the scale of the input attributes is more likely to correspond to the size of the target shrubs.

During the averaging process, the point‐cloud was filtered for ground (Normalized *Z* < 0.15 m), low vegetation (0.15 m > Normalized *Z* < 0.30 m), and high vegetation (Normalized *Z* > 3.5 m) to exclude points without interest to the shrub classification. Yet, the filtered points would have contributed as neighboring points in the attribute computation before the averaging step and therefore should be considered when interpreting the results.

##### Variable selection

This section describes the procedure on which variables have been selected to classify shrubs from two different flights (Autumn 2017 and Spring 2018).

1 Initially, several variables were computed with the OPALS software and each on three different scales (search radius: 0.1, 0.25, and 0.5 m). From these, a set of 17 variables were selected manually to sort out variables identical on the three scales.
2 The 17 “Base” variables were all used for the first classification process to identify their importance in distinguishing shrub genera, and in particularly *C. scoparius*. Two types of models were processed, one containing all shrub classes (All) and one with *C. scoparius* alone from each flight, resulting in a total of 4 models. The importance scores were derived from a “leave‐one‐out” procedure, meaning that each classification was processed firstly with all variables and afterwards one time without each of the variables. In that way, it was possible to calculate the average difference in overall accuracy (OA), completeness and correctness when a variable was not included (Figure A2.3). The OA is measured as the number of correctly classified points, that is, the sum of true positives (TP) and true negatives (TN) divided by the total number of validation points. Additionally, “completeness” is the ratio between TP and TP + false negatives (FN) and “correctness” is the ratio between TP and TP + false positives (FP; Heipke, Mayer, Wiedemann, & Jamet, 1997). Completeness and correctness are equivalent to the terms recall and precision, respectively, also referred to in this document (Forman, 2003). For evaluation, the accuracy measures were summed without weight and ranked from best to worst (Table A2.1).

**TABLE A2.1:** Variable importance based on overall accuracy, completeness and correctness scores. The three measures were summed without additional weights (sum) and ranked from best to worst (1–17) according to each measure before averaging (rank). Color indicates the variables that are kept in the models for the next step (green), and those left out due to lack of importance with a rank threshold ≤ 10 (Red) or autocorrelation with pairwise cor >.75 (yellow)

Variable	Overall accuracy	Completeness	Correctness	Rank	Sum
PseudowaveL	0.047808195	0.00902837	0.047616142	1	0.104453
PDist	0.025160961	0.00811807	0.000653427	5.333333	0.033932
PCountL	0.013440036	0.002558371	0.01761165	3.666667	0.03361
Pcount_ground	0.006272326	0.002858146	0.008466972	5	0.017597
NoOfEchos	0.003692739	0.004184393	0.006445916	6	0.014323
NegOpen	0.004667219	2.81416E−05	0.009419098	7	0.014114
Range	0.004524673	−0.005458463	0.014149932	9	0.013216
NormZ	0.006381884	0.003413731	−9.15274E−05	7.333333	0.009704
Planarity	0.001868897	−0.000228497	0.005266631	10	0.006907
PCountM	0.003716534	−0.000250142	0.003000489	10	0.006467
Rank	0.001781419	0.002352647	0.001707153	10	0.005841
PseudowaveS	0.00252669	0.000889567	0.001788535	9.333333	0.005205
Linearity	0.000436458	0.000820137	0.000593598	12.33333	0.00185
PDensL	0.001645286	0.000281219	−0.000591189	12.66667	0.001335
PCountS	0.001047605	−0.002076538	−0.001163251	14.66667	−0.00219
StdDev	−0.001450466	−0.005435544	0.004469112	13.33333	−0.00242
PosOpen	−0.003849571	−0.003340636	−0.004994585	16.33333	−0.01218

Furthermore, the variables were evaluated in a correlation matrix to avoid autocorrelation. Variable values were extracted from the point cloud to a cell‐based raster and analyzed with R statistics. For extracting the cell values, we chose a search radius of 0.25 m equal to the averaging cylinder used for each variable before the classification. Furthermore, we only extracted values from the classification input points, that is, shrub points. Correlations were made separately for each dataset (Mols17 and Mols18), and the variables were evaluated for both cases. We allowed only variables with maximum pairwise correlation of *ρ* = .75 keeping the most important variable in case of autocorrelation. Variables with importance ranks above 10 or summed importance values below 0.005 were also rejected for further proceeding.

##### Evaluating shrub signatures

We used the shrub locations to extract raster values for the NormZ variable, and each of the seven variables used in the classification around a 25 cm buffer. The samples were compared for each year of data acquisition with a Wilcoxon signed‐rank test to evaluate the differences among signatures in the variables for each shrub species (Table A2.2). *Calluna* and *Pinus* samples were excluded because of limited sample sizes. From the reference data, we see that *Betula* shrubs differ more within the selected variables, while *Crataegus* and *Juniperus* show no signature differences between the years. For the *Cytisus* shrubs, we find a temporal variation in three variables (*p*‐val < .05), namely PCount_ground, PCount, and PDist. However, the NormZ variable used for estimating biomass remains similar for all shrubs except *Betula*. This indicates that our biomass estimations are transferable in time. Furthermore, we would expect that the density variables from *Cytisus* shrubs are most affected by temporal change, as even small leaves will contribute to an increase if there are many. In that sense, we would also have expected more temporal difference in *Crataegus* and *Quercus* shrubs, which have bigger leaves than *Cytisus*. However, it could be explained by temporal variability among species, where the leaves could have been present or not during both flights. Alternatively, the old senescent leaves and additional plant litter from other species growing together with the shrubs may affect the shrub signatures to be interpreted as more equal.

Our findings regarding shrub signatures reveal many new possibilities in terms of understanding vegetation dynamics but would require a much more focused effort for data collection which was beyond the scope of the present study.

#### Final classification results

We classified a total of 59,127,474 points from the 2017 survey and 53,886,091 from 2018. Of these, 12.6% and 6.2% were classified as shrubs for the 2017 and 2018 data, respectively. The rest were generally classified as ground (2017:25.4%; 2018:35.8%), low vegetation (42.8%; 52.0%), and high vegetation (19.3%; 5.9%). From the shrub point cloud, 13 classes could be distinguished from the classification model. The class distribution results are provided as number of points and as percentages (Figure A2.4). For this inventory, each point was assigned the class containing the highest probability value.

#### Classification accuracy assessment

We assessed the accuracy of the final classification input by performing a randomly stratified selection of input training/validation subsets and running 100 permutations for each classification model (2017 and 2018). Furthermore, each model was evaluated with two different splits of training/validation data, that is, 90/10 and 70/30. For each permutation, a confusion matrix was computed showing true positives (TP), true negatives (TN), false positives (FP), and false negatives (FN), whereof the accuracy metrics was derived.

To assess the general classification performance, we calculated the overall accuracy (OA) and Kappa coefficient (Kap) (Fielding & Bell, 1997). The Kappa coefficient compares the observed accuracy with the expected accuracy by random chance. For class‐wise accuracy, we used precision (P) and recall (R) values to calculate the harmonic mean termed “F1” (Forman, 2003). The F1 is calculated by the ratio of (2 * recall * precision) and the sum of recall and precision. For the definition of OA, recall (i.e., completeness) and precision (i.e., correctness) see above. The results from the accuracy assessment are presented in Figure A2.5 for OA and Kappa, while the class‐wise accuracies are shown in Figure A2.6.

Density plots from the 100 permutations of the OA and Kap show the highest accuracy for the 2018 dataset (OA mean = 0.95 and Kap mean = 0.93), while the different ways of splitting training/validation subsets are performing equal. The equal performance of the 90/10 and 70/30 splits was also found for the class‐wise assessment; hence, only results from the 90/10 splits are presented in Figure A2.6.

##### Applied classification model

The final classification models from each year were computed after the 100 validation iterations. For each year, the classification was used to derive biomass metrics in the project area. The resulting accuracy metrics are presented in Table A2.3 and the extraction of biomass metrics in Appendix 3. The final model was selected after the accuracy assessment of the 100 models but with a single similar 90/10 split validation. In a comparison with the mean overall accuracy from the 100 models, the model output used for the biomass projections differed with +0.2% in 2017 and +0.5% in 2018. As the iterative validation process required each model to be overwritten each time, it was not possible to produce a mean or median model output to be applied for biomass extraction. Since the model used for biomass projections differed only minimal to the average of the 100 permutated models and thus shows comparable accuracy, we are confident that no bias has been introduced by applying this model.

**TABLE A2.3:** Accuracy measures from the final models used for calculating biomass from the 2017 and 2018 flights. Accuracies derived from the resulting confusion matrix are presented as overall accuracy and Kappa coefficient. The class‐wise accuracies are presented as precision, recall, and F1 measures

Accuracy assessment—Final model						
	2017	2018				
Overall Accuracy	0.8706	0.956609				
Kappa	0.8394	0.935633				

Class‐wise accuracies						
	Precision		Recall		F1	
	2017	2018	2017	2018	2017	2018
*Cytisus*	0.894	0.967	0.969	0.985	0.930	0.976
*Juniperus*	0.895	0.934	0.802	0.955	0.846	0.945
*Rubus*	0.812	0.941	0.892	0.981	0.850	0.961
*Rosa*	0.860	0.961	0.697	0.956	0.770	0.958
*Quercus*	0.823	0.879	0.706	0.884	0.760	0.881
*Pinus*	0.810	0.955	0.734	0.750	0.770	0.840
Not Shrub	0.884	0.986	0.880	0.839	0.882	0.907
Fencepost	0.923	0.957	0.701	0.941	0.797	0.949
*Sambucus*	0.937	0.914	0.873	0.914	0.904	0.914
*Crataegus*	0.915	0.992	0.628	0.967	0.744	0.979
*Prunus*	0.840	1.000	0.817	0.804	0.828	0.892
*Malus*	0.888	0.960	0.925	0.829	0.906	0.890
*Betula*	0.865	0.933	0.833	0.933	0.849	0.933

##### Merged classification

To assess model transferability, we applied the classification process to a merged point cloud containing both flights (2017 + 2018). Using the mixed signatures from two different seasonal states showed an expected decrease in overall accuracy (Table A2.4). The *Cytisus* class remained high in accuracy compared to the other classes, whereof some would be more affected by seasonal state (e.g., *Betula*) or small sample sizes (*Pinus* = 2 individuals).

**TABLE A2.4:** Accuracy measures from the merged classification including the 2017 and 2018 point clouds. Accuracies derived from the resulting confusion matrix are presented as overall accuracy and Kappa coefficient. The class‐wise accuracies are presented as precision, recall, and F1 measures

Accuracy assessment—Final model						
	70 split	90 split				
Overall Accuracy	0.829	0.832				
Kappa	0.775	0.780				

Class‐wise accuracies						
	Precision		Recall		F1	
	70 split	90 split	70 split	90 split	70 split	90 split
*Cytisus*	0.839	0.843	0.963	0.963	0.963	0.963
*Juniperus*	0.869	0.873	0.784	0.786	0.784	0.786
*Rubus*	0.767	0.770	0.838	0.843	0.838	0.843
*Rosa*	0.768	0.758	0.612	0.625	0.612	0.625
*Quercus*	0.807	0.817	0.617	0.623	0.617	0.623
*Pinus*	0.702	0.701	0.204	0.206	0.204	0.206
Not Shrub	0.940	0.932	0.672	0.680	0.672	0.680
Fencepost	0.962	0.963	0.766	0.787	0.766	0.787
*Sambucus*	0.874	0.877	0.774	0.774	0.774	0.774
*Crataegus*	0.895	0.910	0.667	0.666	0.667	0.666
*Prunus*	0.760	0.759	0.642	0.652	0.642	0.652
*Malus*	0.887	0.911	0.841	0.843	0.841	0.843
*Betula*	0.813	0.819	0.746	0.755	0.746	0.755

#### Additional 2D classification

We performed a traditional 2D classification based on the rasterized variables for comparison with the 3D process. The entire classification was performed with the “caret” package in R statistics using the same GNSS observations as the 3D point cloud classification and the rasterized variables as input data. For this case, we split the reference data in 70% for training and 30% for validation. Besides the recursive partitioning algorithm (Rpart), we tried using a random forest (RF), gradient boosting machines (GBM), and deep neural network (DNN). Each trained model was built using the average resampled estimates from a 10‐fold cross‐validation procedure repeated three times. The classification predictions on the validation set were assessed with overall accuracy and Kappa values (Table A2.4). The resulting overall accuracies did not exceed 50%, and Kappa values were all lower than 0.25.

The 2D classification did not achieve similar good results as the point‐based approach (Table A2.5) and regardless of the classifier used, and the overall accuracy does not exceed 50%. The variables used are somehow modified for a 3D classification but should still be indicative for the different species. The major issue, causing the low performance in the 2D approach, is most probably the reduced information gained from the training data with one less dimension. Therefore, rethinking the creation, assignment, and increasing the amount of training data would be necessary to solve this question from a 2D perspective.

The classification results presented in Table A2.5 suggested a relatively similar performance for the different classifiers. The DNN showed the highest overall accuracy for the 2018 (48.2%) data and lower for 2017 (39.7%), while it failed to deliver Kappa values above 0, possibly because of a lack of training data and/or insufficient preprocessing of the variables. When evaluating both seasons (2017 and 2018), the RF algorithm showed higher overall accuracies (45.6% for 2017 and 42.6% for 2018), and for 2017, it achieved the highest Kappa value (24.7%). The Rpart classifier, similar to the one used for 3D classification, was not markedly worse than the DNN with overall accuracies in 2017 and 2018 of 47.1% and 38.9%, respectively. It can be further noticed that all classifiers show higher accuracies in the leaf‐on period, which is opposite of the 3D classification, that performs better with the leaf‐off data. This again indicates a need for seasonal timing for especially 2D based classifications.

**TABLE A2.5:** Overall accuracy and Kappa values resulting from a 2D raster classification of shrub species. Both datasets from 2017 and 2018 were classified with four different classifiers (Model name)

Model name	Overall accuracy	Kappa
Rpart17	0.4706	0.1915
Rpart18	0.3889	0.1449
RF17	0.4559	0.2465
RF18	0.4259	0.1838
GBM17	0.3971	0.1761
GBM18	0.3889	0.1518
DNN17	0.3971	0
DNN18	0.4815	0

## Appendix 3

### Extracting data for biomass estimation

This document provides information on the digital collection of biomass data recognized through the UAS LiDAR point clouds from Mols with drone flights on 6 October 2017 and 23 April 2018. The aim was to relate the structural volume variables calculated from each individual *Cytisus scoparius* shrub in the point clouds to 10 harvested individuals in various sizes, which have been dried and measured for actual biomass. The individuals were harvested on 2 May 2018 and incubated at 60°C for two days before measuring. We then compared the measures with the 2018 point cloud.

#### Data collection

Before cutting the shrubs, the structure and shape were measured with an RTK GNSS device, resulting in small manually created point clouds of each 25 points (Cyt3D in Figure A3.1). The Cyt3D point clouds were constructed by always measuring top and bottom points first, defined as the most upper splitting of branches and the lowest part measurable on the main stem. The additional 23 points were measured in various heights and distances from the main stem, but always on a branch split or directly on the main stem. This was expected to minimize the inclusion of the smaller, and in wind moving branches, which is possibly not detectable by the UAS LiDAR system. In Figure A3.1, the alignment of the manual constructed point cloud to the LiDAR point cloud is visualized to highlight the precision of the different data sources.

#### Generating and collecting digital biomass data

As described in the classification document (Appendix 2), we classified *C. scoparius* into separate point clouds (i.e., only containing *C. scoparius* points), wherefrom volume metrics based on normalized height could be derived. The normalized height values were each exported as raster formats in 5 cm pixels to represent volume (Figure A3.2). From the 25 manually constructed points, we derived a convex hull area with 25 cm buffer around to delimit the area representing an individual shrub.

Values from each pixel within the defined areas were then extracted and summed for the 10 measured shrubs and used for the biomass models. Three of the harvested shrubs were not revealed in the classified *C. scoparius* LiDAR points*,* but two were instead found among the generally classified low vegetation class and one was classified as Fencepost. These were still included in the biomass models, to evaluate how well we estimate biomass from LiDAR derived point clouds but were not used in the total biomass estimation as it also relies on the ability to classify *C. scoparius*. The measured and extracted values for the 10 individual shrubs are presented in Table A3.1.

**TABLE A3.1:** Values from the 10 harvested individuals by measured dry weight biomass in gram (Weight) and extracted volume in m^3^ from exported raster data (Mean, Max and Range)

Individual	Weight	Mean	Max	Range
1	132	0.01196	0.00786	0.00033
2	430.8	0.07370	0.09342	0.03822
3	626.5	0.13262	0.11979	0.02830
4	33.7	0.00550	0.00555	0.00010
5	104.6	0.01923	0.01183	0.00256
6	287.6	0.03568	0.04844	0.02597
7	48.8	0.00131	0.00131	0.00000
8	153.1	0.01802	0.02552	0.01223
9	554.6	0.03844	0.04625	0.01692
10	107.1	0.00313	0.00365	0.00102

## References

[ece36240-bib-0001] Aplin, P. (2005). Remote sensing: Ecology. Progress in Physical Geography, 29(1), 104–113. 10.1191/030913305pp437pr

[ece36240-bib-0002] Balsi, M. , Esposito, S. , Fallavollita, P. , & Nardinocchi, C. (2018). Single‐tree detection in high‐density LiDAR data from UAV‐based survey. European Journal of Remote Sensing, 51(1), 679–692. 10.1080/22797254.2018.1474722

[ece36240-bib-0003] Bellingham, P. J. (1998). Shrub succession and invasibility in a New Zealand montane grassland. Australian Journal of Ecology, 23, 562–573. 10.1111/j.1442-9993.1998.tb00766.x

[ece36240-bib-0004] Cao, X. , Liu, Y. , Cui, X. , Chen, J. , & Chen, X. (2018). Mechanisms, monitoring and modeling of shrub encroachment into grassland : A review. International Journal of Digital Earth, 1–17, 10.1080/17538947.2018.1478004

[ece36240-bib-0005] Chance, C. M. , Coops, N. C. , Plowright, A. A. , Tooke, T. R. , Christen, A. , & Aven, N. (2016). Invasive shrub mapping in an urban environment from hyperspectral and LiDAR‐derived attributes. Frontiers in Plant Science, 7(OCTOBER2016), 1–19. 10.3389/fpls.2016.01528 27818664 PMC5073150

[ece36240-bib-0006] Chaponnière, P. , & Allouis, T. (2016). The YellowScan Surveyor: 5cm Accuracy Demonstrated Study Site and Dataset. http://www.microgeo.it/public/userfiles/Droni/YellowScanSurveyor_whitePaper_accuracy.pdf

[ece36240-bib-0007] D'Odorico, P. , Okin, G. S. , & Bestelmeyer, B. T. (2012). A synthetic review of feedbacks and drivers of shrub encroachment in arid grasslands. Ecohydrology, 5(5), 520–530. 10.1002/eco.259

[ece36240-bib-0008] Deák, B. , Valkó, O. , Török, P. , & Tóthmérész, B. (2016). Factors threatening grassland specialist plants – A multi‐proxy study on the vegetation of isolated grasslands. Biological Conservation, 204, 255–262. 10.1016/j.biocon.2016.10.023

[ece36240-bib-0009] De'ath, G. , & Fabricius, K. E. (2000). Classification and regression trees: A powerful yet simple technique for ecological data analysis. Ecology, 81(11), 3178–3192.

[ece36240-bib-0010] Donoghue, D. N. M. , Watt, P. J. , Cox, N. J. , & Wilson, J. (2007). Remote sensing of species mixtures in conifer plantations using LiDAR height and intensity data. Remote Sensing of Environment, 110(4), 509–522. 10.1016/j.rse.2007.02.032

[ece36240-bib-0011] Ejrnæs, R. , Bruun, H. H. (2000). Gradient analysis of dry grassland vegetation in Denmark. Journal of Vegetation Science, 11(4), 573–584. 10.2307/3246587

[ece36240-bib-0012] Fick, S. E. , & Hijmans, R. J. (2017). WorldClim 2: New 1‐km spatial resolution climate surfaces for global land areas. International Journal of Climatology, 37(12), 4302–4315. 10.1002/joc.5086

[ece36240-bib-0013] Foody, G. M. (1996). Fuzzy modelling of vegetation from remotely sensed imagery. Ecological Modelling, 85(1), 3–12. 10.1016/0304-3800(95)00012-7

[ece36240-bib-0014] Forman, G. (2003). An extensive empirical study of feature selection. Journal of Machine Learning Research, 3, 1289–1305.

[ece36240-bib-0015] Forsmoo, J. , Anderson, K. , Macleod, C. J. A. , Wilkinson, M. E. , & Brazier, R. (2018). Drone‐based Structure‐from‐Motion photogrammetry captures grassland sward height variability. Journal of Applied Ecology, 55.6, 2587–2599. 10.1111/1365-2664.13148

[ece36240-bib-0016] Haubensak, K. A. , & Parker, I. M. (2004). Soil changes accompanying invasion of the exotic shrub *Cytisus scoparius* in glacial outwash prairies of western Washington [USA]. Plant Ecology, 175(1), 71–79. Retrieved from https://www.jstor.org/stable/20146672

[ece36240-bib-0017] Hellesen, T. , & Matikainen, L. (2013). An object‐based approach for mapping shrub and tree cover on grassland habitats by use of LiDAR and CIR orthoimages. Remote Sensing, 5(2), 558–583. 10.3390/rs5020558

[ece36240-bib-0018] Hernandez‐Santin, L. , Rudge, M. L. , Bartolo, R. E. , & Erskine, P. D. (2019). Identifying species and monitoring understorey from UAS‐derived data: A literature review and future directions. Drones, 3(1), 9 10.3390/drones3010009

[ece36240-bib-0019] Hill, D. A. , Prasad, R. , & Leckie, D. G. (2016). Mapping of Scotch broom (*Cytisus scoparius*) with landsat imagery. Weed Technology, 30(02), 539–558. 10.1614/WT-D-15-00038.1

[ece36240-bib-0020] Kane, V. R. , McGaughey, R. J. , Bakker, J. D. , Gersonde, R. F. , Lutz, J. A. , & Franklin, J. F. (2010). Comparisons between field‐ and LiDAR‐based measures of stand structural complexity. Canadian Journal of Forest Research, 40(4), 761–773. 10.1139/x10-024

[ece36240-bib-0021] Kesting, S. , Petersen, U. , & Isselstein, J. (2015). Humped‐back shaped response of plant species richness to increasing shrub encroachment in calcareous grasslands. Community Ecology, 16(2), 189–195. 10.1556/168.2015.16.2.6

[ece36240-bib-0022] Kukunda, C. B. , Beckschäfer, P. , Magdon, P. , Schall, P. , Wirth, C. , & Kleinn, C. (2019). Scale‐guided mapping of forest stand structural heterogeneity from airborne LiDAR. Ecological Indicators, 102, 410–425. 10.1016/j.ecolind.2019.02.056

[ece36240-bib-0023] Leduc, M. , & Knudby, A. J. (2018). Mapping wild leek through the Forest Canopy using a UAV. Remote Sensing, 10.1(70), 10.3390/rs10010070

[ece36240-bib-0024] Lefsky, M. A. , Cohen, W. B. , Parker, G. G. , & Harding, D. J. (2002). Lidar remote sensing for ecosystem studies. BioScience, 52(1), 19–30. 10.1641/0006-3568(2002)052[0019:LRSFES]2.0.CO;2

[ece36240-bib-0025] Magurran, A. E. (2016). How ecosystems change. Science, 351, 448–449. 10.1126/science.aad6758 26823412

[ece36240-bib-0026] Manfreda, S. , McCabe, M. , Miller, P. , Lucas, R. , Pajuelo Madrigal, V. , Mallinis, G. , … Toth, B. (2018). On the use of unmanned aerial systems for environmental monitoring. Remote Sensing, 10(4), 1–28. 10.3390/rs10040641

[ece36240-bib-0027] Millard, K. , & Richardson, M. (2015). On the importance of training data sample selection in Random Forest image classification: A case study in peatland ecosystem mapping. Remote Sensing, 7(7), 8489–8515. 10.3390/rs70708489

[ece36240-bib-0028] Mitchell, J. J. , Glenn, N. F. , Sankey, T. T. , Derryberry, D. W. R. , Anderson, M. O. , & Hruska, R. C. (2011). Small‐footprint lidar estimations of sagebrush canopy characteristics. Photogrammetric Engineering and Remote Sensing, 77(5), 521–530. 10.14358/PERS.77.5.521

[ece36240-bib-0029] Moeslund, J. E. , Zlinszky, A. , Ejrnæs, R. , Brunbjerg, A. K. , Bøcher, P. K. , Svenning, J.‐C. , & Normand, S. (2019). LIDAR explains diversity of plants, fungi, lichens and bryophytes across multiple habitats and large geographic extent. BioRxiv, 10.1101/509794 PMC6852470 31002436

[ece36240-bib-0030] Müllerová, J. , Brůna, J. , Bartaloš, T. , Dvořák, P. , Vítková, M. , & Pyšek, P. (2017). Timing is important: Unmanned aircraft vs. satellite imagery in plant invasion monitoring. Frontiers Plant Science, 8(May), 1–13. 10.3389/fpls.2017.00887 PMC5449470 28620399

[ece36240-bib-0031] Oldeland, J. , Naftal, L. , & Strohbach, B. J. (2017). The potential of UAV derived image features for discriminating savannah tree species In Díaz‐Delgado R., Lucas R., & Hurford C. (Eds.), The roles of remote sensing in nature conservation (pp. 183–201). Springer, Cham: Springer International Publishing 10.1007/978-3-319-64332-8

[ece36240-bib-0032] Parker, I. M. (2000). Invasion dynamics of *Cytisus scoparius*: A matrix model approach. Ecological Applications, 10(3), 726–743. 10.2307/2641041

[ece36240-bib-0033] Paynter, Q. , Fowler, S. V. , Memmott, J. , & Sheppard, A. W. (1998). Factors affecting the establishment of *Cytisus scoparius* in southern France: Implications for managing both native and exotic populations. Journal of Applied Ecology, 35(4), 582–595.

[ece36240-bib-0034] Peterson, D. J. , & Prasad, R. (1998). The biology of Canadian weeds. 109. *Cytisus scoparius* (L.) Link. Canadian Journal of Plant Science, 78(1961), 497–504. 10.4141/P97-079

[ece36240-bib-0035] Pfeifer, N. , Mandlburger, G. , Otepka, J. , & Karel, W. (2014). OPALS – A framework for Airborne Laser Scanning data analysis. Computers, Environment and Urban Systems, 45, 125–136. 10.1016/j.compenvurbsys.2013.11.002

[ece36240-bib-0036] Popescu, S. (2011). Lidar remote sensing In Weng Q. (Ed.), Advances in environmental remote sensing: Sensors, algorithms, and applications (pp. 57–80). Boca Raton, FL: Taylor & Francis.

[ece36240-bib-0037] QGIS Development Team . (2019). QGIS geographic information system. Retrieved from http://www.qgis.org/

[ece36240-bib-0038] R Core Team . (2016). R: A language and environment for statistical computing. R Foundation for Statistical Computing. Retrieved from https://www.r‐project.org

[ece36240-bib-0039] Ressl, C. , Mandlburger, G. , & Pfeifer, N. (2009). Investigating adjustment of airborne laser scanning strips without usage of GNSS/IMU trajectory data. Laser Scanning 2009, IAPRS, XXXVIII(1), 195–200.

[ece36240-bib-0040] Sanders, A. (2017). Mapping the distribution of understorey rhododendron ponticum using low‐tech multispectra UAV derived imagery In Díaz‐Delgado R., Lucas R., & Hurford C. (Eds.). The roles of remote sensing in nature conservation (pp. 167–181). Chem, Switzerland: Springer.

[ece36240-bib-0041] Sankey, T. , Shrestha, R. , Sankey, J. B. , Hardegree, S. , & Strand, E. (2013). Lidar‐derived estimate and uncertainty of carbon sink in successional phases of woody encroachment. Journal of Geophysical Research: Biogeosciences, 118(3), 1144–1155. 10.1002/jgrg.20088

[ece36240-bib-0042] Sheppard, A. W. , Hodge, P. , Paynter, Q. , & Rees, M. (2002). Factors affecting invasion and persistence of broom *Cytisus scoparius* in Australia. Journal of Applied Ecology, 39(5), 721–734. 10.1046/j.1365-2664.2002.00750.x

[ece36240-bib-0043] Stevens, C. J. , Dise, N. B. , Mountford, J. O. , & Gowing, D. J. (2004). Impact of nitrogen deposition on the species richness of grasslands. Science (New York, N.Y.), 303(5665), 1876–1879. 10.1126/science.1094678 15031507

[ece36240-bib-0044] Svendsen, L. , van der Bijl, L. , Boutrup, S. , & Norup, B. (2005). National monitoring and assessment programme for the aquatic and terrestrial. Aarhus, Denmark: National Environmental Research Institute.

[ece36240-bib-0045] Svenning, J.‐C. , Pedersen, P. B. M. , Donlan, C. J. , Ejrnæs, R. , Faurby, S. , Galetti, M. , … Vera, F. W. M. (2016). Science for a wilder Anthropocene: Synthesis and future directions for trophic rewilding research. Proceedings of the National Academy of Sciences of the United States of America, 113(4), 898–906. 10.1073/pnas.1502556112 26504218 PMC4743824

[ece36240-bib-0046] Therneau, T. M. , & Atkinson, E. J. (1997). An introduction to recursive partitioning using the RPART routines. Rochester, MN: Mayo Foundation.

[ece36240-bib-0047] Timmermann, A. , Damgaard, C. , Strandberg, M. T. , & Svenning, J. C. (2015). Pervasive early 21st‐century vegetation changes across Danish semi‐natural ecosystems: More losers than winners and a shift towards competitive, tall‐growing species. Journal of Applied Ecology, 52(1), 21–30. 10.1111/1365-2664.12374

[ece36240-bib-0048] Valbuena, R. , Eerikäinen, K. , Packalen, P. , & Maltamo, M. (2016). Gini coefficient predictions from airborne lidar remote sensing display the effect of management intensity on forest structure. Ecological Indicators, 60, 574–585. 10.1016/j.ecolind.2015.08.001

[ece36240-bib-0049] Van Aardt, J. A. , Wu, J. , Mcglinchy, J. , Wessels, K. J. , Mathieu, R. S. , Kennedy Bowdoin, T. , … Asner, G. P. (2012). On using discrete return Lidar distributions as a proxy for waveform Lidar signals when modeling vegetation. IEEE International Geoscience and Remote Sensing Symposium (IGARSS), Munich, Germany.

[ece36240-bib-0050] Van Leeuwen, M. , Hilker, T. , Coops, N. C. , Frazer, G. , Wulder, M. A. , Newnham, G. J. , & Culvenor, D. S. (2011). Assessment of standing wood and fiber quality using ground and airborne laser scanning: A review. Forest Ecology and Management, 261(9), 1467–1478. 10.1016/j.foreco.2011.01.032

[ece36240-bib-0051] Vauhkonen, J. (2018). Predicting the provisioning potential of forest ecosystem services using airborne laser scanning data and forest resource maps. Forest Ecosystems, 5(1), 1–19. 10.1186/s40663-018-0143-1

[ece36240-bib-0052] Wachendorf, M. , Fricke, T. , & Möckel, T. (2017). Remote sensing as a tool to assess botanical composition, structure, quantity and quality of temperate grasslands. Grass and Forage Science, 73(1), 1–14. 10.1111/gfs.12312

[ece36240-bib-0053] Wallace, L. , Lucieer, A. , Malenovský, Z. , Turner, D. , & Vopěnka, P. (2016). Assessment of forest structure using two UAV techniques: A comparison of airborne laser scanning and structure from motion (SfM) point clouds. Forests, 7(3), 62 10.3390/f7030062

[ece36240-bib-0054] Wallace, L. , Lucieer, A. , & Watson, C. S. (2014). Evaluating tree detection and segmentation routines on very high resolution UAV LiDAR ata. IEEE Transactions on Geoscience and Remote Sensing, 52(12), 7619–7628. 10.1109/TGRS.2014.2315649

[ece36240-bib-0055] Wallace, L. , Lucieer, A. , Watson, C. , & Turner, D. (2012). Development of a UAV‐LiDAR system with application to forest inventory. Remote Sensing, 4(6), 1519–1543. 10.3390/rs4061519

[ece36240-bib-0056] Wang, D. , Xin, X. , Shao, Q. , Brolly, M. , Zhu, Z. , & Chen, J. (2017). Modeling aboveground biomass in Hulunber grassland ecosystem by using unmanned aerial vehicle discrete LiDAR. Sensors, 17(12), 1–19. 10.3390/s17010180 PMC5298753 28106819

[ece36240-bib-0057] Wieser, M. , Mandlburger, G. , Hollaus, M. , Otepka, J. , Glira, P. , & Pfeifer, N. (2017). A case study of UAS borne laser scanning for measurement of tree stem diameter. Remote Sensing, 9(11), 1154 10.3390/rs9111154

[ece36240-bib-0058] Wijesingha, J. , Moeckel, T. , Hensgen, F. , & Wachendorf, M. (2019). Evaluation of 3D point cloud‐based models for the prediction of grassland biomass. International Journal of Applied Earth Observation and Geoinformation, 78(May 2018), 352–359. 10.1016/j.jag.2018.10.006

[ece36240-bib-0059] Wilsey, B. J. (2018). Conservation and restoration of grasslands (Chapter 8) In Wilsey B. J. (Ed.), The biology of grasslands (pp. 143–163). Oxford, UK: Oxford University Press 10.1093/oso/9780198744511.001.0001

[ece36240-bib-0060] YellowScan . (2016). YellowScan Surveyor – User Manual. https://www.yellowscan‐lidar.com/products/surveyor/

[ece36240-bib-0061] Yokoyama, R. , Shirasawa, M. , & Pike, R. J. (2002). Visualizing topography by openness: A new application of image processing to digital elevation models. Photogrammetric Engineering & Remote Sensing, 68(3), 257–265. 10.1016/j.jtbi.2012.08.013

[ece36240-bib-0062] Zlinszky, A. , & Kania, A. (2016). Will it blend? Visualization and accuracy evaluation of high‐resolution fuzzy vegetation maps. International Archives of the Photogrammetry, Remote Sensing and Spatial Information Sciences – ISPRS Archives, 41(July), 335–342. 10.5194/isprsarchives-XLI-B2-335-2016

[ece36240-bib-0063] Zlinszky, A. , Schroiff, A. , Kania, A. , Deák, B. , Mücke, W. , Vári, Á. , … Pfeifer, N. (2014). Categorizing grassland vegetation with full‐waveform airborne laser scanning: A feasibility study for detecting natura 2000 habitat types. Remote Sensing, 6(9), 8056–8087. 10.3390/rs6098056

